# Exploring *Tenebrio molitor* as a source of low‐molecular‐weight antimicrobial peptides using a n *in silico* approach: correlation of molecular features and molecular docking

**DOI:** 10.1002/jsfa.13949

**Published:** 2024-10-16

**Authors:** Teresa Gonzalez‐de la Rosa, Elvira Marquez‐Paradas, Maria J Leon, Sergio Montserrat‐de la Paz, Fernando Rivero‐Pino

**Affiliations:** ^1^ Department of Medical Biochemistry, Molecular Biology, and Immunology School of Medicine, University of Seville Seville Spain; ^2^ Instituto de Biomedicina de Sevilla, IBiS, Hospital Universitario Virgen del Rocío, CSIC University of Seville Seville Spain; ^3^ Department of Microbiology and Parasitology School of Pharmacy, University of Seville Seville Spain; ^4^ European Food Safety Authority, Nutrition and Food Innovation Unit, Novel Foods Team Parma Italy

**Keywords:** bacteria, bioactive peptides, fungi, peptidome, proteomic profile, virus

## Abstract

**BACKGROUND:**

Yellow mealworm (*Tenebrio molitor*) larvae are increasingly recognized as a potential source of bioactive peptides due to their high protein content. Antimicrobial peptides from sustainable sources are a research topic of interest. This study aims to characterize the peptidome of *T*. *molitor* flour and an Alcalase‐derived hydrolysate, and to explore the potential presence of antimicrobial peptides using *in silico* analyses, including prediction tools, molecular docking and parameter correlations.

**RESULTS:**

*T*. *molitor* protein was hydrolysed using Alcalase, resulting in a hydrolysate (TMH10A) with a 10% degree of hydrolysis. The peptidome was analysed using LC‐TIMS‐MS/MS, yielding over 6000 sequences. These sequences were filtered using the PeptideRanker tool, selecting the top 100 sequences with scores >0.8. Bioactivity predictions indicated that specific peptides, particularly WLNSKGGF and GFIPYEPFLKKMMA, showed significant antimicrobial potential, particularly against bacteria, fungi and viruses. Correlations were found between antifungal activity and physicochemical properties such as net charge, hydrophobicity and isoelectric point.

**CONCLUSIONS:**

The study identified specific *T*. *molitor*‐derived peptides with strong predicted antimicrobial activity through *in silico* analysis. These peptides, particularly WLNSKGGF and GFIPYEPFLKKMMA, might offer potential applications in food safety and healthcare. Further experimental validation is required to confirm their efficacy. © 2024 The Author(s). *Journal of the Science of Food and Agriculture* published by John Wiley & Sons Ltd on behalf of Society of Chemical Industry.

## INTRODUCTION

In recent years, the use of insects as food has grown exponentially in Western societies because the safety and nutritional composition that they have are being described and, considering their environmentally friendly production process, it is attracting the interest of the food industry as well as of consumers. The dried yellow mealworm (*Tenebrio molitor*) was the first species to obtain authorization in the European Union,^1^, ^2^ and consequently research on the benefits that its consumption might exert is being carried out in order to fully explore and exploit this insect as food. From a nutritional perspective, eating insects is interesting because of their high protein content (ranging around 450–600 g kg^−1^) and amino acid content (high in leucine, valine and lysine, among others), fatty acid profile (250–400 g kg^−1^ of fat, consisting primarily of unsaturated fatty acids), vitamin and mineral content, and other ingredients like chitin (50–150 g kg^−1^). Additionally, studies on the bioactive peptides that are encrypted in some insect species' protein sequences remain in progress. These peptides may have a variety of functions that are relevant to human health.^3^

For *T*. *molitor* peptides obtained through enzymatic hydrolysis, bioactivities such antioxidant, antidiabetic,^4^ antithrombotic^5^ or anti‐inflammatory^6^ have been described by numerous studies *in vitro*,^7^ in which rarely was identification of sequences carried out. In fact, according to Teixeira *et al*.,^8^ only 27 sequences have been identified in *T*. *molitor*, from which only the activity of a few of them was evaluated by *in vitro* or *in vivo* means with synthetic peptides, mostly regarding antioxidant and anti‐inflammatory peptides.^9^, ^10^ Consequently, there is a gap in research concerning the peptidome of *T*. *molitor* protein hydrolysates obtained by different enzymatic treatments, knowledge of which could help to unravel the mechanisms by which these pools of peptides exert certain bioactivities. One of the most interesting bioactivities that peptides might exert is antimicrobial activity, which can be exerted through different mechanisms and targeting several types of microorganisms including bacteria, fungi or viruses.^11^ Antimicrobial peptides (AMPs) from *T*. *molitor* obtained through enzymatic hydrolysis of its meal or protein isolate have been scarcely studied.^12^

Insect products have numerous prospective applications as functional ingredients in food. Nonetheless, to properly utilize such products and enhance their usage to benefit human health, there are still a lot of challenges and gaps in research to overcome.^12^ The structural characterization of unidentified components derived from *T*. *molitor* and their functionality require further study, including the peptidome and the contribution of each sequence to the bioactivity of protein hydrolysates, which can be estimated by means of *in silico* tools.^13^ We hypothesize that subjecting a high‐protein‐content (around 450 g kg^−1^)^13^ *T*. *molitor* flour to an enzymatic treatment with Alcalase yielding a degree of hydrolysis of 10% (as demonstrated for other sources^7^, ^8^, ^11^, ^12^) will produce a pool of peptides with antimicrobial character, since the protease can release different peptides in terms of length (8–20 amino acids) and position of amino acids in the chain (high proportion of cationic residues and high proportion of hydrophobic amino acids).^8^, ^12^

Antimicrobial protein hydrolysates have been reported for different insects (*Omphisa fuscidentalis*, *Musca domestica*, *Tenebrio molitor*, *Bombyx mori*, *Gryllus* sp., *Locusta migratoria* and *Oecophylla smaragdina*), obtained by simulated gastrointestinal enzymatic digestion, but no identification of sequences was reported.^14^ Similarly, Flores *et al*.^15^ assessed the antimicrobial properties of protein hydrolysates from *Tenebrio molitor* and *Ulomoides dermestoides*, but no identification of the sequences was carried out. In relation to *in silico* analyses, Fu *et al*.^16^ employed bioinformatics analysis to identify peptides with antimicrobial activity from *Protaetia brevitarsis* Lewis larvae, suggesting that the sequences VFRLKKWIQKVI, KIYVLLRRQA and STLHLVLRLR‐NH2 could be effective against *Candida albicans*, *Escherichia coli* and *Staphylococcus aureus*. *In silico* research on the potential of edible insects as sources of peptides against SARS‐CoV‐2 has been also reported. PKWF and VHRKCF were proposed as adequate antiviral peptides from *T*. *molitor*, which could target SARS‐CoV‐2 spike glycoprotein.^17^

Concerning the mechanisms by which peptides can exert antimicrobial activity, it must be noted that cell membranes present a negative charge, and consequently cationic and amphipathic peptides are able to interact with them, potentially disrupting them, leading to eventual death of a cell. On top of that, some peptides are able to enter inside the cells, and then impact the intracellular processes occurring, which could also lead to cell death.^18^, ^19^ It is known that hydrophobic residues enter bacterial membranes, which will cause their disruption, cytoplasmic leakage and ultimately cell death.^20^ For antifungal peptides, on top of cell membrane disruption, their interaction with specific lipids, reactive oxygen species production, autophagy or programmed cell death are also mechanisms to exert activity.^21^ Regarding the growth of viruses, it might be inhibited by targeting transcription, attachment and interaction with specific proteins. Nonetheless, considering the diversity of viruses, the characterization of peptides able to inhibit them is challenging, mostly because of the mechanisms by which the antiviral activity could be exerted, which has widely been associated with destabilization of membranes. In this context, *in silico* analysis can help in the study of interactions between compounds. Recently it was shown that secondary structural integrity, hydrophobic properties and surface charge contribute much to antimicrobial activities.^22^ The aim of the work reported here was to characterize the peptidome of *T*. *molitor* flour and a hydrolysate obtained with Alcalase, and to unravel through *in silico* analyses (i.e. prediction tools, molecular docking and correlation of parameters) the potential presence of AMPs.

## MATERIALS AND METHODS

### Hydrolysis of *T*. *molitor* flour

The hydrolysis was carried out in a jacketed reactor under continuous stirring at constant temperature (50 °C) and pH 8, resuspending *Tenebrio molitor* flour with *ca* 450 g kg^−1^ of protein content (TMF, InsektLabel Biotech, Biscay, Spain) at 28 g of protein L^−1^ in distilled water and adding Alcalase 2.4 L (Novozymes, Bagsvaerd, Denmark) at an enzyme/substrate (E/S) ratio of 0.375%. Once a degree of hydrolysis of 10 was achieved (measured by the pH‐stat method), enzyme deactivation for the sample was achieved by freezing at −80 °C and then the sample (TMH10A) was freeze‐dried and stored until analysis.^13^

### Peptide extraction, purification and sequence identification by LC‐TIMS‐MS/MS

The samples (TMF and TMH10A) were acidified with 0.5% (v/v) trifluoroacetic acid. Desalting and concentration step was performed with ZipTip C18 (Millipore) and the samples were speed‐vacuum‐dried. LC‐TIMS‐MS/MS was carried out using a nanoElute nanoflow ultrahigh‐pressure LC system (Bruker Daltonics, Bremen, Germany) coupled to a timsTOF Pro 2 mass spectrometer, equipped with a CaptiveSpray nanoelectrospray ion source (Bruker Daltonics) according to the procedure described in the patent P202230873. Further details of the procedure can be found in Montserrat‐de la Paz *et al*.^23^ The reference library was acquired from UniProt_proteome_Tenebrio‐molitor_Jan24 and protein unique peptides were set to larger than 1 and a high confidence score of −10lgP > 17.5 was applied to indicate an accurately identified protein. Sequences of up to 50 residues were identified.

### *In silico* analysis

#### Prediction tools

As the amount of identified sequences was high (6856 and 14 994, respectively, for TMF and TMH10A), a first filter was applied in order to reduce the number of peptides to be included in the *in silico* analyses. In this regard, PeptideRanker, which ranks sequences by predicted probability to be bioactive,^24^ assigning a score ranging from 0 to 1.0, was used to filter the first 100 sequences having >0.8 score. This analysis resulted in 91 sequences in TMF and 100 sequences in TMH10A. All these peptides (*n* = 190, since one was found in both samples) were subjected to the following prediction tools: (i) ToxinPred software was used to predict, for each peptide, its hydrophobicity, side bulk, amphipathicity, hydrophilicity, hydropathicity, steric hindrance, toxicity, net hydrogen, isoelectric point, charge and molecular weight^25^; (ii) to estimate the likelihood of being antimicrobial using different algorithms, the tools CAMPR3,^26^ Antimicrobial Peptide Scanner vr.2^27^ and Macrel^28^ were employed; and (iii) iAMPpred was used to estimate the likelihood of being specifically antibacterial, antiviral or antifungal.^29^

#### *In silico* correlation of parameters

Prior to statistical analysis, the dataset was preprocessed, eliminating peptides that appeared more than once. In the search for possible correlations of interest, the calculation of Pearson's coefficient was used as a linear correlation value between variables. Correlation heat maps were constructed using the ggplot2 package (v3.4.2) from the correlation matrix between peptide characteristics and peptide antifungal, antiviral and antibacterial capacity.^30^ To test whether some variables could be good predictors of the levels of others, i.e. whether they could act as significant explanatory variables, the most relevant correlations observed were fitted by linear regression models. The coefficient of determination (*R* ^2^) was used to estimate the overall quality of the fit.

#### Molecular docking

Molecular docking was carried out to determinate the binding affinity energy of several peptides with different bacterial, viral and fungal receptors. The selected bacterial receptors were oxygen‐insensitive NADPH nitroreductase from *Helicobacter pylori* (RdxA, PDB: 3QDL), glucose‐1‐phosphate thymidylyltransferase from *Pseudomonas aeruginosa* (RmIA, PDB: 4ASJ), penicillin‐binding protein 2a from *Staphylococcus aureus* subsp. *aureus* Mu50 (PBP2A, PDB: 4CJN) and penicillin‐binding protein 3 from *Pseudomonas aeruginosa* (PBP3, PDB: 4KQQ); the viral receptor was COVID‐19 main protease in the apo state (PDB: 7C2Q); and the selected fungal receptor was lanosterol 14‐*α* demethylase from *Saccharomyces cerevisiae* (PDB: 4LXJ). The X‐ray crystal structures of these receptors were obtained from the RCSB PDB database (Protein Data Bank, http://www.rcsb.org/). Ligands and all the water molecules were removed from each receptor PDB file, while polar hydrogen atoms were added using UCSF Chimera software. The three‐dimensional structures of the peptides were obtained, and their structure was minimized by USCF Chimera and prepared for docking simulation. Docking analyses were done with different peptides for each receptor. In the case of the bacterial receptors, the simulation was performed with the peptides GGGGGLGGGGGL and WLNSKGGF; for the viral receptor the selected peptides were GFIPYEPFLKKMMA and FSNPIFRIF; and the peptides analysed with the fungal receptor were GFIPYEPFLKKMMA and FGPKGVGFGMGAGALTMA. Then, the molecular structures of receptors and peptides were converted to PDBQT format with AutoDock Tools. AGFR program was used to calculate the positions and sizes of the specific docking boxes for each receptor–peptide complex. Finally, the potential best docking score determined was selected and visualized via Biovia Discovery Studio Visualizer, as well as the two‐dimensional (2D) and surface annotation of both ligand interactions with the protein.

## RESULTS AND DISCUSSION

### Peptidome profile of *T*. *molitor* flour and hydrolysates

The peptidome for the original flour and the hydrolysate was fully characterized by LC‐TIMS‐MS/MS. The number of peptides identified in each sample was 6856 for TMF and 14 994 for TMH10A, indicating how the protein hydrolysis released several low‐molecular‐weight peptides, as there was an increase of 54% in absolute numbers of identified sequences. From the raw peptidome from each sample, it is difficult to draw global conclusions, since the relative amount and the number of peptides identified are highly variable. On top of that, TMF is expected to contain proteins whose molecular weight can vary from 14 to 100 kDa,^31^ which would not be identified in this analysis. For this reason, the difference in the amount of low‐molecular‐weight peptides is not high between samples, in terms of percentage, since in TMF 2627 identified peptides had a length of up to 9 amino acids (38.3%), whereas in TMH10A the number was 6047 (40.3%), although when filtering up to 20 amino acids, 6419 peptides in TMH (93.6%) and 14 309 (95.4%) were identified. It must be noted that the hydrolysis was done up to a degree of hydrolysis of 10%, which is not an extensive hydrolysis; thus, the amount of low‐molecular‐weight peptides similar between samples is reasonable. However, only 563 peptides were identified in both samples, indicating that the hydrolysis by Alcalase was effective in releasing other peptides, and it could have increased their bioactivity, since the samples contain different sequences except for these 563 sequences. Alcalase is a broad‐spectrum endopeptidase which will increase the amount of N‐terminal locations available.^32^ As indicated previously, the identification of *T*. *molitor* peptides is still a gap in research, since the identification of these is not commonly reported in the literature; so, for the first time, to the authors’ knowledge, a full characterization of the peptidome by LC–MS was carried out, helping to unravel the potentially bioactive compounds in the samples. Given the abundance of components found in protein hydrolysates (such as free amino acids, small‐ and medium‐sized peptides, polypeptides, oligomers, undigested proteins, etc.), it is difficult and limited to accurately characterize every single peptide in the hydrolysate, and not cost‐effective to chemically synthesize all of them. It might be challenging to discern between large‐molecular‐weight chemicals when they are present and low‐molecular‐weight peptides (four amino acids in length), which are frequently those responsible for the bioactivity. Thus, bioinformatic analysis plays a vital role in the discovery of physiologically active peptides. As indicated in the methodology, a first filter employing the widely used PeptideRanker tool was done in order to focus the *in silico* assessment on those whose likelihood to be bioactive is high (score > 0.8), as indicated previously,^24^ and from this filter, a maximum of 100 sequences were computed and collected for further analyses.

### *In silico* evaluation of peptidomes

#### Physicochemical and bioactivity characterization

In Table 1, the physical–chemical properties (hydrophobicity, side bulk, amphipathicity, hydrophilicity, hydropathicity, steric hindrance, toxicity, net hydrogen, isoelectric point, charge and molecular weight) are presented. In the case of the outcome from the prediction tools related to bioactivity (likelihood to be bioactive and the antimicrobial potential by different tools), the results are presented in Table 2. Based on the analysis carried out with ToxinPred software, all the peptides were predicted as non‐toxic, except for IDIPPPNGPLW (from the TMH sample). As can be observed, only 91 peptides from those identified in the TMH sample had a PeptideRanker score >0.8, whereas in the TMH10A, 529 peptides were found to have at least that score, although in Tables 1 and 2 only the first 100 sequences are depicted, consequently becoming the threshold 0.912. This is a first indication that hydrolysis of *T*. *molitor* meal could ameliorate the bioactivity of the sample, as several authors have reported, in relation to different bioactivities.^7^, ^9^, ^12^ This behaviour in which a hydrolysed protein might be more bioactive than the native protein is due to the exposure of residues that might interact with different targets, whereas, when encrypted in the native protein, due to structural hindrance, those sequences are unable to interact.

**Table 1:** Characterization of the most bioactive peptides from the peptidome according to the PeptideRanker prediction tool, identified in TMF and TMH10A, based on *in silico* analyses in relation to physicochemical properties

	PeptideRanker^a^	ToxinPred^b^										
		Toxicity	Hydrophobicity	Steric hindrance	Sidebulk	Hydropathicity	Amphipathicity	Hydrophilicity	Net hydrogen	Charge	pI	Molecular weight
Sequences: TMF												
SWDWGPAF	0.975	Non‐Toxin	0.09	0.57	0.57	−0.44	0.00	−0.81	0.50	−1.00	3.80	965.13
GFQPDLF	0.950	Non‐Toxin	0.06	0.63	0.63	0.06	0.18	−0.51	0.43	−1.00	3.80	823.01
GPAGYLRPW	0.949	Non‐Toxin	−0.05	0.56	0.56	−0.57	0.27	−0.56	0.67	1.00	9.10	1016.29
WGPAWNINM	0.946	Non‐Toxin	0.09	0.62	0.62	−0.29	0.00	−1.11	0.67	0.00	5.88	1088.38
IDIPPPNGPLW	0.936	Toxin	0.08	0.55	0.55	−0.17	0.00	−0.51	0.36	−1.00	3.80	1218.58
FGGPEMW	0.919	Non‐Toxin	0.12	0.63	0.63	−0.30	0.18	−0.60	0.29	−1.00	4.00	823.03
NWFIDRL	0.918	Non‐Toxin	−0.13	0.66	0.66	−0.19	0.35	−0.47	1.14	0.00	6.19	963.20
AGYLRPW	0.917	Non‐Toxin	−0.07	0.57	0.57	−0.44	0.35	−0.71	0.86	1.00	9.10	862.09
EDWAGRFWP	0.915	Non‐Toxin	−0.16	0.60	0.60	−1.19	0.41	−0.09	0.89	−1.00	4.38	1163.37
NAGQFPF	0.911	Non‐Toxin	0.03	0.63	0.63	−0.23	0.18	−0.73	0.57	0.00	5.88	779.94
NWGPEWGL	0.910	Non‐Toxin	0.03	0.59	0.59	−0.93	0.16	−0.67	0.62	−1.00	4.00	958.16
AKDLPPLGF	0.909	Non‐Toxin	0.01	0.57	0.57	0.13	0.41	−0.07	0.33	0.00	6.19	957.26
GFIPYEPFLKKMMA	0.905	Non‐Toxin	0.03	0.63	0.63	0.24	0.61	−0.36	0.43	1.00	8.83	1672.28
DHPGWMKL	0.904	Non‐Toxin	−0.12	0.54	0.54	−0.98	0.64	−0.13	0.62	0.50	7.09	983.27
FGPKGVGFGMGAGALTMA	0.901	Non‐Toxin	0.18	0.63	0.63	0.79	0.20	−0.54	0.17	1.00	9.11	1669.26
SGSFLSPWA	0.896	Non‐Toxin	0.12	0.54	0.54	0.34	0.00	−0.81	0.44	0.00	5.88	951.16
HGGTNWGFL	0.895	Non‐Toxin	0.09	0.56	0.56	−0.32	0.16	−0.93	0.56	0.50	7.10	988.21
ESHPGFW	0.893	Non‐Toxin	−0.03	0.49	0.49	−1.09	0.39	−0.44	0.57	−0.50	5.25	859.00
GGDWERF	0.890	Non‐Toxin	−0.26	0.67	0.67	−1.49	0.53	0.44	1.00	−1.00	4.38	865.99
DHPGWMK	0.888	Non‐Toxin	−0.21	0.54	0.54	−1.66	0.73	0.11	0.71	0.50	7.09	870.09
AAPYGYGGLG	0.887	Non‐Toxin	0.16	0.60	0.60	0.16	0.00	−0.74	0.20	0.00	5.87	925.15
NSWGSGWG	0.878	Non‐Toxin	0.01	0.61	0.61	−1.01	0.00	−0.75	0.75	0.00	5.88	849.97
AGPGHWNDPDML	0.876	Non‐Toxin	−0.07	0.56	0.56	−0.92	0.12	−0.11	0.50	−1.50	4.20	1309.59
DGFIPYEPFLKKMMA	0.872	Non‐Toxin	−0.02	0.64	0.64	−0.01	0.57	−0.13	0.47	0.00	6.41	1787.38
ADPLNGF	0.871	Non‐Toxin	0.02	0.62	0.62	−0.09	0.00	−0.23	0.43	−1.00	3.80	732.88
NWGPEWG	0.868	Non‐Toxin	−0.04	0.59	0.59	−1.60	0.18	−0.51	0.71	−1.00	4.00	844.98
SGGDWERF	0.868	Non‐Toxin	−0.26	0.65	0.65	−1.40	0.47	0.43	1.00	−1.00	4.38	953.08
MGWMHWE	0.866	Non‐Toxin	0.06	0.56	0.56	−0.73	0.39	−0.99	0.57	−0.50	5.25	976.24
DGRPWWE	0.865	Non‐Toxin	−0.32	0.59	0.59	−2.19	0.53	0.31	1.14	−1.00	4.38	945.09
GWPNPDLA	0.864	Non‐Toxin	−0.02	0.56	0.56	−0.74	0.00	−0.31	0.50	−1.00	3.80	869.05
WSLIDNF	0.863	Non‐Toxin	0.09	0.64	0.64	0.34	0.00	−0.86	0.71	−1.00	3.80	894.09
ADPINGF	0.862	Non‐Toxin	0.05	0.64	0.64	0.01	0.00	−0.23	0.43	−1.00	3.80	732.88
DDGFIPYEPFLKKMMA	0.860	Non‐Toxin	−0.06	0.65	0.65	−0.23	0.54	0.06	0.50	−1.00	4.56	1902.48
AEEDWAGRFW	0.860	Non‐Toxin	−0.17	0.62	0.62	−1.08	0.50	0.17	0.90	−2.00	4.14	1266.46
GSYKPHIYGF	0.857	Non‐Toxin	−0.01	0.57	0.57	−0.56	0.51	−0.61	0.60	1.50	8.84	1168.46
GFADGARPF	0.857	Non‐Toxin	−0.06	0.62	0.62	−0.13	0.27	0.00	0.56	0.00	6.19	937.13
FLQPQGF	0.855	Non‐Toxin	0.07	0.62	0.62	0.06	0.36	−0.91	0.57	0.00	5.88	836.06
HGGTNWGF	0.855	Non‐Toxin	0.03	0.57	0.57	−0.84	0.18	−0.82	0.62	0.50	7.10	875.03
DNFEWGL	0.854	Non‐Toxin	−0.04	0.66	0.66	−0.74	0.18	−0.21	0.71	−2.00	3.67	880.02
DGRPWWERY	0.853	Non‐Toxin	−0.45	0.62	0.62	−2.34	0.69	0.32	1.44	0.00	6.42	1264.48
SGSNVFSMF	0.852	Non‐Toxin	0.08	0.66	0.66	0.60	0.00	−0.74	0.56	0.00	5.88	975.20
WSDDSFNRP	0.851	Non‐Toxin	−0.38	0.62	0.62	−1.81	0.27	0.43	1.22	−1.00	4.21	1123.25
GGTNWGFLN	0.851	Non‐Toxin	0.06	0.65	0.65	−0.36	0.00	−0.86	0.67	0.00	5.88	965.17
SPPPYSDLGKR	0.851	Non‐Toxin	−0.33	0.56	0.56	−1.47	0.56	0.50	0.91	1.00	8.93	1216.50
DGRPWWER	0.851	Non‐Toxin	−0.50	0.60	0.60	−2.48	0.77	0.65	1.50	0.00	6.42	1101.29
ESHPHFW	0.849	Non‐Toxin	−0.11	0.40	0.40	−1.49	0.60	−0.51	0.71	0.00	6.01	939.09
GWGLTDGF	0.848	Non‐Toxin	0.14	0.63	0.63	0.04	0.00	−0.64	0.38	−1.00	3.80	852.03
HPTWGMLH	0.848	Non‐Toxin	0.03	0.42	0.42	−0.54	0.36	−0.99	0.50	1.00	7.26	978.26
SDWGDNGIFK	0.847	Non‐Toxin	−0.14	0.68	0.68	−0.96	0.37	0.18	0.80	−1.00	4.21	1138.34
AEEDWAGRFWPR	0.846	Non‐Toxin	−0.29	0.61	0.61	−1.41	0.62	0.39	1.08	−1.00	4.68	1519.79
APFTYAAF	0.845	Non‐Toxin	0.22	0.57	0.57	0.92	0.00	−1.15	0.25	0.00	5.88	887.09
ASTDFWKG	0.842	Non‐Toxin	−0.11	0.61	0.61	−0.70	0.46	−0.06	0.75	0.00	6.19	911.08
WLNSKGGF	0.842	Non‐Toxin	−0.02	0.63	0.63	−0.41	0.46	−0.53	0.75	1.00	9.11	908.14
SDNLNGGFL	0.840	Non‐Toxin	−0.03	0.66	0.66	−0.19	0.00	−0.27	0.67	−1.00	3.80	936.12
FGDPIIRP	0.840	Non‐Toxin	−0.05	0.62	0.62	0.02	0.31	−0.01	0.62	0.00	6.19	914.18
AGQFPFAA	0.840	Non‐Toxin	0.17	0.58	0.58	0.69	0.16	−0.79	0.25	0.00	5.88	808.00
TMFPDFY	0.840	Non‐Toxin	0.08	0.65	0.65	0.06	0.00	−0.86	0.43	−1.00	3.80	920.13
DNFEWGLG	0.839	Non‐Toxin	−0.02	0.66	0.66	−0.70	0.16	−0.19	0.62	−2.00	3.67	937.09
EDPDWWK	0.838	Non‐Toxin	−0.36	0.61	0.61	−2.54	0.71	0.74	1.00	−2.00	4.03	975.11
GQFPWQA	0.837	Non‐Toxin	−0.01	0.59	0.59	−0.76	0.36	−0.86	0.71	0.00	5.88	833.01
NAGQFPFA	0.837	Non‐Toxin	0.06	0.61	0.61	0.02	0.16	−0.70	0.50	0.00	5.88	851.03
SHDDLGWL	0.836	Non‐Toxin	−0.06	0.54	0.54	−0.59	0.18	−0.15	0.62	−1.50	4.20	942.11
PFMPDQL	0.835	Non‐Toxin	−0.02	0.60	0.60	−0.24	0.18	−0.34	0.43	−1.00	3.80	847.09
SSNPAGF	0.834	Non‐Toxin	−0.03	0.58	0.58	−0.36	0.00	−0.31	0.57	0.00	5.88	678.78
LIDDHFLF	0.834	Non‐Toxin	0.15	0.59	0.59	0.94	0.18	−0.61	0.38	−1.50	4.20	1019.28
DPDWWKHAT	0.834	Non‐Toxin	−0.24	0.51	0.51	−1.82	0.57	0.09	0.89	−0.50	5.22	1155.35
GGYPGIL	0.831	Non‐Toxin	0.24	0.62	0.62	0.60	0.00	−0.84	0.14	0.00	5.88	675.89
DMPDQPPVWDAAFR	0.831	Non‐Toxin	−0.18	0.60	0.60	−0.84	0.26	0.18	0.71	−2.00	3.94	1645.00
DDTFWIG	0.831	Non‐Toxin	0.04	0.66	0.66	−0.24	0.00	−0.30	0.57	−2.00	3.57	852.99
SWNEDWGDKGYF	0.831	Non‐Toxin	−0.20	0.66	0.66	−1.65	0.41	0.08	0.92	−2.00	4.03	1503.71
SYKPHIYGF	0.830	Non‐Toxin	−0.03	0.56	0.56	−0.58	0.57	−0.68	0.67	1.50	8.84	1111.39
DFQDLFN	0.829	Non‐Toxin	−0.15	0.70	0.70	−0.66	0.18	−0.06	0.86	−2.00	3.57	898.03
DGSYKPHIYGF	0.829	Non‐Toxin	−0.08	0.59	0.59	−0.83	0.47	−0.28	0.64	0.50	7.09	1283.56
MGWLHWE	0.828	Non‐Toxin	0.10	0.52	0.52	−0.46	0.39	−1.06	0.57	−0.50	5.25	958.21
LVADGRPWW	0.826	Non‐Toxin	−0.04	0.58	0.58	−0.22	0.27	−0.51	0.78	0.00	6.19	1099.38
ADNQFPWQ	0.826	Non‐Toxin	−0.20	0.62	0.62	−1.49	0.31	−0.35	1.00	−1.00	3.80	1005.16
SGGSDPDPF	0.822	Non‐Toxin	−0.13	0.60	0.60	−1.09	0.00	0.46	0.44	−2.00	3.57	877.97
KVPVLPFPL	0.820	Non‐Toxin	0.16	0.55	0.55	1.12	0.41	−0.68	0.22	1.00	9.11	1009.43
GFLPNILR	0.819	Non‐Toxin	0.01	0.62	0.62	0.61	0.31	−0.59	0.75	1.00	10.11	929.25
DFQDLFNR	0.815	Non‐Toxin	−0.35	0.70	0.70	−1.14	0.46	0.33	1.25	−1.00	4.21	1054.23
PFTYAAF	0.813	Non‐Toxin	0.21	0.58	0.58	0.80	0.00	−1.24	0.29	0.00	5.88	816.00
EEDPDWWKHATF	0.813	Non‐Toxin	−0.24	0.56	0.56	−1.72	0.64	0.36	0.83	−2.50	4.31	1560.80
VRDHPGWM	0.813	Non‐Toxin	−0.20	0.56	0.56	−1.00	0.49	−0.09	0.88	0.50	7.10	997.25
FYDDWTR	0.813	Non‐Toxin	−0.34	0.66	0.66	−1.66	0.35	0.06	1.29	−1.00	4.21	1002.13
ANWGPEWGLLE	0.812	Non‐Toxin	0.04	0.58	0.58	−0.48	0.23	−0.43	0.55	−2.00	3.80	1271.56
VRDHPGWMKL	0.810	Non‐Toxin	−0.22	0.57	0.57	−0.81	0.76	0.05	0.90	1.50	9.10	1238.62
AEEDWAGRFWP	0.809	Non‐Toxin	−0.16	0.60	0.60	−1.13	0.45	0.15	0.82	−2.00	4.14	1363.59
SSWGPSHPELL	0.808	Non‐Toxin	−0.03	0.48	0.48	−0.55	0.25	−0.33	0.55	−0.50	5.25	1209.48
AAPPIGP	0.803	Non‐Toxin	0.17	0.50	0.50	0.41	0.00	−0.40	0.00	0.00	5.88	621.82
PDWWKHAT	0.801	Non‐Toxin	−0.19	0.48	0.48	−1.61	0.64	−0.27	0.88	0.50	7.09	1040.25
YGMVGFR	0.800	Non‐Toxin	−0.00	0.70	0.70	0.33	0.35	−0.66	0.71	1.00	9.10	829.08
Sequences: TMH10A												
GGGGGIGGGIGGGIGGGIGGGIGGGIGGGL	0.992	Non‐Toxin	0.29	0.68	0.68	0.72	0.00	−0.42	0.00	0.00	5.88	2122.87
GPFMPGF	0.990	Non‐Toxin	0.24	0.61	0.61	0.50	0.00	−0.90	0.00	0.00	5.88	751.99
GYGPFMPGF	0.984	Non‐Toxin	0.20	0.63	0.63	0.20	0.00	−0.96	0.11	0.00	5.88	972.25
SQFWFGFPQRF	0.983	Non‐Toxin	−0.05	0.63	0.63	−0.36	0.45	−0.88	0.91	1.00	10.11	1446.78
QFWFGFPQRFMLPK	0.979	Non‐Toxin	−0.04	0.62	0.62	−0.21	0.62	−0.72	0.79	2.00	11.01	1829.40
GDGGLGGGIGGGHGGGIGGGIGGGIGGGIGGGHGGGIGGGIGGGH	0.978	Non‐Toxin	0.20	0.64	0.64	0.20	0.10	−0.29	0.09	0.50	6.28	3333.33
GWFVNPF	0.973	Non‐Toxin	0.23	0.63	0.63	0.49	0.00	−1.39	0.43	0.00	5.88	866.08
GGGFGGGHGGGGGGGFGGGF	0.973	Non‐Toxin	0.20	0.65	0.65	−0.06	0.07	−0.40	0.05	0.50	7.10	1509.85
GEFPWMM	0.973	Non‐Toxin	0.14	0.64	0.64	0.03	0.18	−0.79	0.29	−1.00	4.00	897.17
WWDSPLLRPF	0.972	Non‐Toxin	−0.05	0.55	0.55	−0.34	0.25	−0.66	0.80	0.00	6.19	1316.66
DNSFFGGF	0.972	Non‐Toxin	0.07	0.69	0.69	−0.03	0.00	−0.50	0.50	−1.00	3.80	890.02
FNPPGFAF	0.971	Non‐Toxin	0.18	0.60	0.60	0.39	0.00	−0.97	0.25	0.00	5.88	896.11
FGAPVFF	0.970	Non‐Toxin	0.39	0.62	0.62	1.77	0.00	−1.36	0.00	0.00	5.88	784.01
SQFWFGFPQ	0.969	Non‐Toxin	0.07	0.61	0.61	−0.26	0.28	−1.13	0.67	0.00	5.88	1143.39
GGPFGGGPF	0.968	Non‐Toxin	0.21	0.61	0.61	0.04	0.00	−0.56	0.00	0.00	5.88	791.99
SDFADFF	0.968	Non‐Toxin	0.05	0.67	0.67	0.34	0.00	−0.24	0.43	−2.00	3.57	847.95
SDFFPGDF	0.967	Non‐Toxin	0.03	0.65	0.65	−0.18	0.00	−0.15	0.38	−2.00	3.57	931.06
AYFGFPR	0.966	Non‐Toxin	−0.03	0.62	0.62	−0.06	0.35	−0.69	0.71	1.00	9.10	857.06
SVPSFGSEWFWFR	0.966	Non‐Toxin	0.00	0.60	0.60	−0.12	0.29	−0.68	0.77	0.00	6.36	1631.98
RFPIFFH	0.965	Non‐Toxin	0.05	0.55	0.55	0.51	0.56	−0.97	0.71	1.50	10.11	963.24
QFPWQAF	0.964	Non‐Toxin	0.06	0.59	0.59	−0.30	0.36	−1.21	0.71	0.00	5.88	923.13
AFGAPVFF	0.963	Non‐Toxin	0.37	0.61	0.61	1.77	0.00	−1.25	0.00	0.00	5.88	855.10
FGFDGDFFGR	0.962	Non‐Toxin	−0.03	0.70	0.70	−0.15	0.25	−0.10	0.60	−1.00	4.21	1164.37
KAAGGGGGIGGGIGGGIGGGIGGGIGGGIGGGL	0.960	Non‐Toxin	0.24	0.67	0.67	0.65	0.11	−0.32	0.06	1.00	9.11	2393.24
FSWDWGPA	0.959	Non‐Toxin	0.09	0.57	0.57	−0.44	0.00	−0.81	0.50	−1.00	3.80	965.13
GDGGLGGGIGGGHGGGIGGGIGGGIGGGIGGGH	0.958	Non‐Toxin	0.20	0.64	0.64	0.21	0.09	−0.27	0.09	0.00	5.99	2456.18
AYFGFPRRFLLPK	0.957	Non‐Toxin	−0.11	0.60	0.60	−0.00	0.66	−0.38	0.85	3.00	11.01	1612.13
AFWSGPL	0.956	Non‐Toxin	0.23	0.55	0.55	0.67	0.00	−1.13	0.29	0.00	5.88	776.98
FIDNIFRF	0.956	Non‐Toxin	0.02	0.71	0.71	0.74	0.31	−0.61	0.88	0.00	6.19	1071.35
AAPMFDMM	0.954	Non‐Toxin	0.14	0.65	0.65	0.88	0.00	−0.55	0.12	−1.00	3.80	913.23
QAGAWLPF	0.952	Non‐Toxin	0.18	0.56	0.56	0.47	0.16	−1.06	0.38	0.00	5.88	889.13
DKMPWFKGW	0.950	Non‐Toxin	−0.14	0.63	0.63	−1.16	0.82	−0.18	0.78	1.00	8.94	1194.54
ADQLFRMF	0.950	Non‐Toxin	−0.11	0.67	0.67	0.20	0.46	−0.30	0.88	0.00	6.19	1027.31
FSNPIFRIF	0.950	Non‐Toxin	0.06	0.65	0.65	0.78	0.27	−0.84	0.78	1.00	10.11	1140.47
GPFMPGFS	0.950	Non‐Toxin	0.17	0.60	0.60	0.34	0.00	−0.75	0.12	0.00	5.88	839.08
FQPSFLGM	0.949	Non‐Toxin	0.14	0.62	0.62	0.62	0.16	−0.95	0.38	0.00	5.88	926.21
GWPLDKF	0.949	Non‐Toxin	−0.03	0.60	0.60	−0.53	0.52	−0.24	0.57	0.00	6.19	862.09
LGSGWPF	0.949	Non‐Toxin	0.21	0.57	0.57	0.36	0.00	−1.06	0.29	0.00	5.88	762.96
SFQEFPPLGRF	0.948	Non‐Toxin	−0.09	0.60	0.60	−0.34	0.45	−0.25	0.73	0.00	6.36	1324.65
GGFGGDLGGGHGGFSGGF	0.948	Non‐Toxin	0.15	0.63	0.63	0.02	0.08	−0.36	0.17	−0.50	5.09	1539.87
AIWMGPL	0.947	Non‐Toxin	0.32	0.58	0.58	1.30	0.00	−1.26	0.14	0.00	5.88	787.09
FIADHPFIF	0.946	Non‐Toxin	0.26	0.57	0.57	1.21	0.16	−1.01	0.22	−0.50	5.09	1106.41
WGPEWGL	0.946	Non‐Toxin	0.13	0.56	0.56	−0.56	0.18	−0.80	0.43	−1.00	4.00	844.04
SDRFGFL	0.945	Non‐Toxin	−0.12	0.65	0.65	0.03	0.35	−0.07	0.86	0.00	6.19	841.02
PQGPFAF	0.944	Non‐Toxin	0.11	0.57	0.57	0.04	0.18	−0.76	0.29	0.00	5.88	762.95
GGGGGFGGGF	0.944	Non‐Toxin	0.25	0.68	0.68	0.24	0.00	−0.50	0.00	0.00	5.88	768.94
GGGFGGGF	0.944	Non‐Toxin	0.27	0.69	0.69	0.40	0.00	−0.62	0.00	0.00	5.88	654.80
KESQFWFGFPQRF	0.943	Non‐Toxin	−0.17	0.64	0.64	−0.88	0.76	−0.28	1.00	1.00	9.10	1704.10
QFSYLPW	0.942	Non‐Toxin	0.07	0.57	0.57	−0.21	0.18	−1.36	0.71	0.00	5.88	940.16
GSGWGWLGY	0.940	Non‐Toxin	0.19	0.61	0.61	−0.19	0.00	−1.18	0.44	0.00	5.88	982.20
QIPRLLGPGLNKAGKFPGLL	0.940	Non‐Toxin	−0.03	0.59	0.59	0.12	0.55	−0.22	0.60	3.00	11.17	2089.88
GEFPWMIG	0.937	Non‐Toxin	0.20	0.64	0.64	0.30	0.16	−0.75	0.25	−1.00	4.00	936.21
ADMKHWPF	0.936	Non‐Toxin	−0.10	0.54	0.54	−0.83	0.64	−0.27	0.62	0.50	7.09	1031.30
EAYFGFPRRFLLPK	0.936	Non‐Toxin	−0.15	0.61	0.61	−0.25	0.70	−0.14	0.86	2.00	10.00	1741.26
ANWGPEWGLL	0.936	Non‐Toxin	0.10	0.57	0.57	−0.18	0.13	−0.77	0.50	−1.00	4.00	1142.43
QEFPPLGRF	0.936	Non‐Toxin	−0.14	0.60	0.60	−0.63	0.55	−0.07	0.78	0.00	6.36	1090.37
FLWGPAL	0.936	Non‐Toxin	0.34	0.55	0.55	1.33	0.00	−1.43	0.14	0.00	5.88	803.07
VPFPRLHFFMPGFAPL	0.936	Non‐Toxin	0.14	0.54	0.54	0.76	0.24	−0.90	0.31	1.50	10.11	1873.52
GGGGGLGGGGGLGGGGGLGGGK	0.935	Non‐Toxin	0.15	0.66	0.66	0.01	0.17	−0.11	0.09	1.00	9.11	1512.99
SDKMPWFKGW	0.934	Non‐Toxin	−0.15	0.62	0.62	−1.12	0.73	−0.13	0.80	1.00	8.94	1281.63
SAPIWQF	0.933	Non‐Toxin	0.13	0.57	0.57	0.33	0.18	−1.10	0.57	0.00	5.88	848.06
YSPYFPL	0.931	Non‐Toxin	0.11	0.55	0.55	−0.00	0.00	−1.23	0.43	0.00	5.87	886.10
GWAEPIRFL	0.931	Non‐Toxin	0.02	0.59	0.59	0.22	0.41	−0.44	0.67	0.00	6.36	1088.40
SGPFGQIF	0.931	Non‐Toxin	0.16	0.63	0.63	0.42	0.16	−0.79	0.38	0.00	5.88	852.07
GGGGGLGGGGGL	0.929	Non‐Toxin	0.22	0.65	0.65	0.30	0.00	−0.30	0.00	0.00	5.88	815.06
SSPDLNFF	0.929	Non‐Toxin	−0.02	0.61	0.61	−0.10	0.00	−0.38	0.62	−1.00	3.80	926.09
LPDQWDWRL	0.928	Non‐Toxin	−0.24	0.59	0.59	−1.20	0.41	−0.13	1.11	−1.00	4.21	1228.50
GQFPWMVAL	0.926	Non‐Toxin	0.22	0.61	0.61	0.90	0.14	−1.20	0.33	0.00	5.88	1048.40
QAAFPAPF	0.925	Non‐Toxin	0.14	0.54	0.54	0.54	0.16	−0.79	0.25	0.00	5.88	848.06
SDKDKFFYF	0.924	Non‐Toxin	−0.23	0.69	0.69	−0.94	0.82	0.28	0.89	0.00	6.30	1196.43
FAFFDNL	0.924	Non‐Toxin	0.18	0.67	0.67	1.00	0.00	−0.94	0.43	−1.00	3.80	873.06
GEWYPDHHFRLL	0.924	Non‐Toxin	−0.15	0.51	0.51	−0.97	0.55	−0.32	0.83	0.00	6.02	1569.92
SNPPLWPY	0.923	Non‐Toxin	−0.02	0.51	0.51	−0.94	0.00	−0.88	0.62	0.00	5.88	973.20
ADKGFLW	0.923	Non‐Toxin	0.01	0.62	0.62	−0.04	0.52	−0.31	0.57	0.00	6.19	836.05
GGGYGGGLGGGF	0.923	Non‐Toxin	0.22	0.67	0.67	0.14	0.00	−0.55	0.08	0.00	5.88	955.19
SFSNPIFRIF	0.923	Non‐Toxin	0.03	0.64	0.64	0.62	0.25	−0.73	0.80	1.00	10.11	1227.56
KDWLDSPWSGF	0.922	Non‐Toxin	−0.10	0.59	0.59	−0.88	0.33	−0.14	0.73	−1.00	4.21	1337.60
SAGGLIGAGGLIGTGGLIGAR	0.922	Non‐Toxin	0.18	0.62	0.62	0.99	0.12	−0.45	0.29	1.00	10.11	1768.39
FMPGFAPL	0.922	Non‐Toxin	0.29	0.58	0.58	1.19	0.00	−1.07	0.00	0.00	5.88	879.19
IADHPFIF	0.921	Non‐Toxin	0.22	0.56	0.56	1.01	0.18	−0.82	0.25	−0.50	5.09	959.22
DARPLPEWFDKAKIGIF	0.921	Non‐Toxin	−0.11	0.62	0.62	−0.31	0.65	0.19	0.71	0.00	6.47	2003.57
FGGPEMW	0.919	Non‐Toxin	0.12	0.63	0.63	−0.30	0.18	−0.60	0.29	−1.00	4.00	823.03
PLPQGPF	0.919	Non‐Toxin	0.06	0.52	0.52	−0.30	0.18	−0.59	0.29	0.00	5.88	754.98
WFNPSIL	0.918	Non‐Toxin	0.18	0.58	0.58	0.61	0.00	−1.29	0.57	0.00	5.88	876.12
SNPIFRIF	0.918	Non‐Toxin	−0.01	0.64	0.64	0.52	0.31	−0.64	0.88	1.00	10.11	993.28
SSLFSPW	0.917	Non‐Toxin	0.09	0.53	0.53	0.24	0.00	−0.97	0.57	0.00	5.88	823.00
ASDKMPWFKGW	0.917	Non‐Toxin	−0.11	0.61	0.61	−0.85	0.67	−0.16	0.73	1.00	8.94	1352.72
PDQWDWRLY	0.917	Non‐Toxin	−0.30	0.61	0.61	−1.77	0.41	−0.19	1.22	−1.00	4.21	1278.51
GPAPLQPLPKL	0.917	Non‐Toxin	−0.01	0.51	0.51	−0.09	0.45	−0.25	0.36	1.00	9.11	1130.56
PSPPGGPL	0.916	Non‐Toxin	0.04	0.48	0.48	−0.53	0.00	−0.19	0.12	0.00	5.88	720.93
AVPIPPRFG	0.916	Non‐Toxin	0.04	0.56	0.56	0.40	0.27	−0.37	0.44	1.00	10.11	953.27
GWDDPVLADPLKRKIPLRRF	0.916	Non‐Toxin	−0.28	0.61	0.61	−0.66	0.73	0.45	1.00	2.00	9.99	2393.12
GGGFGGGAGGGF	0.916	Non‐Toxin	0.20	0.63	0.63	0.19	0.08	−0.53	0.11	0.50	7.10	1481.84
DKMPWFK	0.915	Non‐Toxin	−0.25	0.64	0.64	−1.30	1.05	0.26	0.86	1.00	8.94	951.24
SGSFLSPW	0.915	Non‐Toxin	0.10	0.54	0.54	0.16	0.00	−0.85	0.50	0.00	5.88	880.07
NADHPFIF	0.914	Non‐Toxin	0.05	0.56	0.56	0.01	0.18	−0.57	0.50	−0.50	5.09	960.16
WTGIGFM	0.914	Non‐Toxin	0.30	0.65	0.65	0.97	0.00	−1.34	0.29	0.00	5.88	811.07
AQFGFDGDFFGR	0.914	Non‐Toxin	−0.06	0.69	0.69	−0.27	0.31	−0.11	0.67	−1.00	4.21	1363.61
APVWAPGL	0.914	Non‐Toxin	0.24	0.52	0.52	0.89	0.00	−0.96	0.12	0.00	5.88	810.07
PLPQGPFAF	0.912	Non‐Toxin	0.14	0.54	0.54	0.28	0.14	−0.79	0.22	0.00	5.88	973.26

All these tools were accessed in February 2024.

^a^ The likelihood for the peptides as bioactive was evaluated by PeptideRanker (http://bioware.ucd.ie/~compass/biowareweb), a server to predict bioactive peptides based on an *N*‐to‐1 neural network, by giving scores ranging from 0 to 1. Higher score indicates the greater the likelihood of the peptide being bioactive.

^b^ Peptides were subjected to calculation via https://webs.iiitd.edu.in/raghava/toxinpred/design.php/, where the hydrophobicity, steric hinderance and amphipathicity were calculated.

**Table 2:** Characterization of most bioactive peptides from the peptidome according to the PeptideRanker prediction tool, identified in TMF and TMH10A, based on *in silico* analyses in relation to the antimicrobial activity

	CAMPR3^a^							iAMPpred^b^			Antimicrobial Peptide Scanner vr.2^c^		Macrel^d^
	Results with support vector machine (SVM) classifier		Results with random forest classifier		Results with artificial neural network classifier	Results with discriminant analysis classifier		Antibacterial	Antiviral	Antifungal			AMP probability
Sequences: TMF													
SWDWGPAF	NAMP	0.036	NAMP	0.361	AMP	NAMP	0.064	0.713	0.572	0.236	AMP^e^	0.814	0.069
GFQPDLF	NAMP	0.037	NAMP	0.264	AMP	NAMP	0.040	0.348	0.184	0.216	Non‐AMP^e^	0.444	0.079
GPAGYLRPW	NAMP	0.104	NAMP	0.357	AMP	NAMP	0.105	0.204	0.093	0.255	AMP^e^	0.910	0.040
WGPAWNINM	NAMP	0.002	NAMP	0.265	AMP	NAMP	0.254	0.697	0.528	0.277	AMP^e^	0.870	0.040
IDIPPPNGPLW	NAMP	0.006	NAMP	0.391	AMP	NAMP	0.221	0.339	0.287	0.196	Non‐AMP	0.241	0.079
FGGPEMW	AMP	0.789	NAMP	0.470	AMP	NAMP	0.003	0.665	0.582	0.393	Non‐AMP^e^	0.139	0.059
NWFIDRL	NAMP	0.019	NAMP	0.278	AMP	NAMP	0.005	0.363	0.658	0.145	AMP^e^	0.710	0.089
AGYLRPW	NAMP	0.274	NAMP	0.294	AMP	NAMP	0.046	0.425	0.237	0.334	AMP^e^	0.790	0.139
EDWAGRFWP	NAMP	0.000	NAMP	0.333	AMP	NAMP	0.018	0.631	0.630	0.121	AMP^e^	0.584	0.069
NAGQFPF	NAMP	0.352	NAMP	0.382	AMP	NAMP	0.066	0.536	0.401	0.366	AMP^e^	0.511	0.030
NWGPEWGL	NAMP	0.021	NAMP	0.316	AMP	NAMP	0.035	0.747	0.794	0.281	Non‐AMP^e^	0.400	0.069
AKDLPPLGF	NAMP	0.038	NAMP	0.402	AMP	NAMP	0.018	0.267	0.272	0.171	Non‐AMP^e^	0.440	0.089
GFIPYEPFLKKMMA	AMP	0.959	NAMP	0.232	AMP	NAMP	0.057	0.726	0.843	0.812	Non‐AMP	0.157	0.366
DHPGWMKL	AMP	0.678	AMP	0.633	AMP	NAMP	0.010	0.650	0.515	0.354	Non‐AMP^e^	0.349	0.188
FGPKGVGFGMGAGALTMA	AMP	0.943	AMP	0.645	AMP	AMP	0.975	0.717	0.699	0.805	AMP	0.824	0.446
SGSFLSPWA	NAMP	0.020	NAMP	0.160	AMP	NAMP	0.058	0.330	0.052	0.109	AMP^e^	0.936	0.099
HGGTNWGFL	NAMP	0.028	NAMP	0.195	AMP	NAMP	0.015	0.750	0.564	0.460	AMP^e^	0.674	0.158
ESHPGFW	AMP	1.000	NAMP	0.362	NAMP	NAMP	0.010	0.716	0.332	0.240	AMP^e^	0.542	0.079
GGDWERF	NAMP	0.000	NAMP	0.350	AMP	NAMP	0.000	0.320	0.354	0.108	Non‐AMP^e^	0.196	0.257
DHPGWMK	AMP	1.000	AMP	0.661	AMP	NAMP	0.024	0.686	0.540	0.409	Non‐AMP^e^	0.431	0.168
AAPYGYGGLG	NAMP	0.020	NAMP	0.264	AMP	NAMP	0.220	0.354	0.258	0.354	AMP	0.704	0.109
NSWGSGWG	NAMP	0.049	NAMP	0.309	NAMP	NAMP	0.030	0.745	0.442	0.363	AMP^e^	0.699	0.099
AGPGHWNDPDML	NAMP	0.008	NAMP	0.207	NAMP	NAMP	0.009	0.269	0.196	0.127	Non‐AMP	0.014	0.050
DGFIPYEPFLKKMMA	AMP	0.880	NAMP	0.164	NAMP	NAMP	0.029	0.597	0.667	0.613	Non‐AMP	0.072	0.158
ADPLNGF	NAMP	0.024	NAMP	0.397	AMP	NAMP	0.018	0.154	0.285	0.283	Non‐AMP^e^	0.471	0.059
NWGPEWG	NAMP	0.035	NAMP	0.303	AMP	NAMP	0.022	0.736	0.767	0.374	Non‐AMP^e^	0.363	0.099
SGGDWERF	NAMP	0.003	NAMP	0.354	NAMP	NAMP	0.000	0.190	0.150	0.056	Non‐AMP^e^	0.108	0.040
MGWMHWE	NAMP	0.016	NAMP	0.412	NAMP	NAMP	0.001	0.646	0.626	0.435	Non‐AMP^e^	0.114	0.248
DGRPWWE	NAMP	0.000	NAMP	0.293	NAMP	NAMP	0.000	0.740	0.722	0.365	Non‐AMP^e^	0.123	0.149
GWPNPDLA	NAMP	0.001	NAMP	0.367	AMP	NAMP	0.011	0.219	0.351	0.123	AMP^e^	0.509	0.129
WSLIDNF	NAMP	0.133	NAMP	0.279	NAMP	NAMP	0.015	0.446	0.803	0.228	AMP^e^	0.548	0.109
ADPINGF	NAMP	0.042	NAMP	0.427	AMP	NAMP	0.028	0.397	0.321	0.286	Non‐AMP^e^	0.254	0.059
DDGFIPYEPFLKKMMA	AMP	0.756	NAMP	0.170	NAMP	NAMP	0.052	0.480	0.422	0.359	Non‐AMP	0.032	0.149
AEEDWAGRFW	NAMP	0.024	NAMP	0.258	NAMP	NAMP	0.004	0.543	0.557	0.125	Non‐AMP	0.062	0.139
GSYKPHIYGF	NAMP	0.091	NAMP	0.251	AMP	NAMP	0.471	0.428	0.568	0.419	Non‐AMP	0.234	0.109
GFADGARPF	NAMP	0.003	NAMP	0.334	AMP	NAMP	0.117	0.098	0.156	0.128	Non‐AMP^e^	0.480	0.059
FLQPQGF	NAMP	0.041	NAMP	0.355	AMP	NAMP	0.245	0.584	0.194	0.323	Non‐AMP^e^	0.337	0.069
HGGTNWGF	NAMP	0.051	NAMP	0.203	AMP	NAMP	0.009	0.776	0.619	0.490	AMP^e^	0.599	0.188
DNFEWGL	AMP	1.000	NAMP	0.301	NAMP	NAMP	0.011	0.365	0.688	0.163	Non‐AMP^e^	0.191	0.168
DGRPWWERY	NAMP	0.001	NAMP	0.332	NAMP	NAMP	0.002	0.702	0.458	0.362	Non‐AMP^e^	0.406	0.119
SGSNVFSMF	AMP	0.816	NAMP	0.247	AMP	NAMP	0.007	0.553	0.313	0.334	AMP^e^	0.786	0.228
WSDDSFNRP	NAMP	0.000	NAMP	0.324	AMP	NAMP	0.008	0.265	0.190	0.148	Non‐AMP^e^	0.315	0.079
GGTNWGFLN	NAMP	0.000	NAMP	0.297	AMP	NAMP	0.102	0.622	0.568	0.461	AMP^e^	0.775	0.218
SPPPYSDLGKR	AMP	1.000	NAMP	0.221	NAMP	NAMP	0.036	0.232	0.031	0.116	AMP	0.746	0.178
DGRPWWER	NAMP	0.001	NAMP	0.300	NAMP	NAMP	0.001	0.721	0.534	0.283	Non‐AMP^e^	0.222	0.158
ESHPHFW	AMP	1.000	NAMP	0.362	NAMP	NAMP	0.019	0.672	0.600	0.430	Non‐AMP^e^	0.231	0.119
GWGLTDGF	NAMP	0.002	NAMP	0.271	AMP	NAMP	0.011	0.276	0.557	0.106	AMP^e^	0.547	0.149
HPTWGMLH	NAMP	0.034	AMP	0.518	NAMP	NAMP	0.002	0.608	0.771	0.373	AMP^e^	0.610	0.267
SDWGDNGIFK	NAMP	0.033	NAMP	0.241	NAMP	NAMP	0.003	0.691	0.512	0.138	Non‐AMP	0.03	0.109
AEEDWAGRFWPR	NAMP	0.060	NAMP	0.334	AMP	NAMP	0.044	0.253	0.282	0.052	Non‐AMP	0.079	0.089
APFTYAAF	NAMP	0.207	NAMP	0.471	AMP	NAMP	0.122	0.330	0.417	0.195	Non‐AMP^e^	0.439	0.040
ASTDFWKG	NAMP	0.001	NAMP	0.318	AMP	NAMP	0.013	0.475	0.283	0.067	AMP^e^	0.672	0.040
WLNSKGGF	NAMP	0.372	NAMP	0.31	AMP	NAMP	0.444	0.872	0.581	0.366	AMP^e^	0.692	0.050
SDNLNGGFL	NAMP	0.030	NAMP	0.285	NAMP	NAMP	0.046	0.400	0.311	0.661	Non‐AMP^e^	0.091	0.020
FGDPIIRP	NAMP	0.000	NAMP	0.383	AMP	NAMP	0.123	0.642	0.362	0.450	Non‐AMP^e^	0.466	0.040
AGQFPFAA	NAMP	0.241	NAMP	0.384	AMP	NAMP	0.354	0.555	0.372	0.520	AMP^e^	0.651	0.010
TMFPDFY	AMP	0.999	NAMP	0.468	NAMP	NAMP	0.002	0.533	0.442	0.317	Non‐AMP^e^	0.280	0.089
DNFEWGLG	AMP	0.955	NAMP	0.265	NAMP	NAMP	0.001	0.353	0.665	0.157	Non‐AMP^e^	0.140	0.208
EDPDWWK	AMP	1.000	NAMP	0.432	NAMP	AMP	0.822	0.656	0.587	0.407	Non‐AMP^e^	0.057	0.109
GQFPWQA	NAMP	0.005	NAMP	0.291	AMP	NAMP	0.053	0.431	0.124	0.163	AMP^e^	0.723	0.040
NAGQFPFA	NAMP	0.150	NAMP	0.369	AMP	NAMP	0.140	0.546	0.412	0.435	Non‐AMP^e^	0.481	0.020
SHDDLGWL	NAMP	0.001	NAMP	0.365	NAMP	NAMP	0.002	0.549	0.511	0.215	Non‐AMP^e^	0.099	0.059
PFMPDQL	AMP	0.636	NAMP	0.492	NAMP	NAMP	0.004	0.656	0.335	0.508	Non‐AMP^e^	0.441	0.129
SSNPAGF	AMP	1.000	NAMP	0.239	NAMP	NAMP	0.259	0.357	0.219	0.277	Non‐AMP^e^	0.439	0.188
LIDDHFLF	AMP	0.686	NAMP	0.457	AMP	NAMP	0.162	0.607	0.399	0.349	Non‐AMP^e^	0.060	0.188
DPDWWKHAT	NAMP	0.127	NAMP	0.367	NAMP	NAMP	0.116	0.654	0.494	0.311	Non‐AMP^e^	0.458	0.079
GGYPGIL	NAMP	0.000	NAMP	0.271	AMP	NAMP	0.261	0.693	0.576	0.482	AMP^e^	0.528	0.050
DMPDQPPVWDAAFR	NAMP	0.073	NAMP	0.079	NAMP	NAMP	0.006	0.142	0.091	0.082	Non‐AMP	0.075	0.149
DDTFWIG	AMP	0.986	NAMP	0.384	NAMP	NAMP	0.001	0.587	0.500	0.232	Non‐AMP^e^	0.175	0.050
SWNEDWGDKGYF	NAMP	0.012	NAMP	0.231	NAMP	NAMP	0.003	0.672	0.462	0.135	Non‐AMP	0.006	0.059
SYKPHIYGF	AMP	0.897	NAMP	0.246	NAMP	AMP	0.605	0.301	0.500	0.293	Non‐AMP^e^	0.251	0.168
DFQDLFN	AMP	0.974	NAMP	0.311	NAMP	NAMP	0.013	0.547	0.461	0.444	Non‐AMP^e^	0.037	0.139
DGSYKPHIYGF	NAMP	0.134	NAMP	0.130	AMP	NAMP	0.076	0.229	0.288	0.296	Non‐AMP	0.050	0.089
MGWLHWE	NAMP	0.001	NAMP	0.396	NAMP	NAMP	0.004	0.684	0.644	0.417	Non‐AMP^e^	0.062	0.317
LVADGRPWW	NAMP	0.004	NAMP	0.365	NAMP	NAMP	0.031	0.637	0.742	0.116	Non‐AMP^e^	0.218	0.089
ADNQFPWQ	NAMP	0.003	NAMP	0.384	NAMP	NAMP	0.181	0.392	0.248	0.169	Non‐AMP^e^	0.022	0.030
SGGSDPDPF	NAMP	0.000	AMP	0.504	NAMP	NAMP	0.006	0.249	0.163	0.190	Non‐AMP^e^	0.263	0.228
KVPVLPFPL	NAMP	0.310	AMP	0.587	AMP	AMP	0.625	0.653	0.684	0.654	AMP^e^	0.718	0.287
GFLPNILR	NAMP	0.025	NAMP	0.382	AMP	AMP	0.780	0.756	0.644	0.748	AMP^e^	0.927	0.238
DFQDLFNR	NAMP	0.009	NAMP	0.310	NAMP	NAMP	0.011	0.416	0.355	0.360	Non‐AMP^e^	0.043	0.149
PFTYAAF	NAMP	0.018	NAMP	0.430	AMP	NAMP	0.034	0.278	0.405	0.144	AMP^e^	0.502	0.089
EEDPDWWKHATF	NAMP	0.269	NAMP	0.155	NAMP	NAMP	0.030	0.514	0.262	0.113	Non‐AMP	0.011	0.050
VRDHPGWM	NAMP	0.005	NAMP	0.419	NAMP	NAMP	0.000	0.505	0.411	0.171	AMP^e^	0.609	0.129
FYDDWTR	NAMP	0.002	NAMP	0.350	NAMP	NAMP	0.000	0.567	0.437	0.265	Non‐AMP^e^	0.074	0.109
ANWGPEWGLLE	NAMP	0.003	NAMP	0.322	NAMP	NAMP	0.041	0.531	0.770	0.100	Non‐AMP	0.007	0.139
VRDHPGWMKL	NAMP	0.134	NAMP	0.304	NAMP	NAMP	0.001	0.370	0.412	0.118	Non‐AMP	0.314	0.188
AEEDWAGRFWP	NAMP	0.009	NAMP	0.258	AMP	NAMP	0.011	0.379	0.448	0.074	Non‐AMP	0.034	0.010
SSWGPSHPELL	NAMP	0.091	NAMP	0.176	NAMP	NAMP	0.001	0.089	0.051	0.035	Non‐AMP	0.056	0.158
AAPPIGP	NAMP	0.005	AMP	0.530	AMP	NAMP	0.087	0.234	0.232	0.224	AMP^e^	0.624	0.297
PDWWKHAT	NAMP	0.024	NAMP	0.344	NAMP	NAMP	0.091	0.727	0.689	0.362	AMP^e^	0.861	0.069
YGMVGFR	NAMP	0.124	NAMP	0.266	NAMP	NAMP	0.003	0.573	0.578	0.581	AMP^e^	0.701	0.178
Sequences: TMH10A													
GGGGGIGGGIGGGIGGGIGGGIGGGIGGGL	AMP	0.916	AMP	0.757	AMP	AMP	0.999	0.839	0.627	0.629	AMP	0.999	0.564
GPFMPGF	AMP	0.989	AMP	0.559	AMP	NAMP	0.227	0.721	0.586	0.633	AMP^e^	0.694	0.040
GYGPFMPGF	AMP	0.635	AMP	0.544	AMP	NAMP	0.156	0.607	0.495	0.567	AMP^e^	0.704	0.089
SQFWFGFPQRF	NAMP	0.027	NAMP	0.273	AMP	NAMP	0.031	0.649	0.309	0.393	AMP	0.953	0.109
QFWFGFPQRFMLPK	NAMP	0.138	NAMP	0.415	AMP	NAMP	0.033	0.805	0.551	0.699	AMP	0.799	0.228
GDGGLGGGIGGGHGGGIGGGIGGGIGGGIGGGHGGGIGGGIGGGH	AMP	0.998	AMP	0.658	AMP	AMP	0.999	0.891	0.713	0.744	AMP	0.999	0.396
GWFVNPF	NAMP	0.011	NAMP	0.423	AMP	NAMP	0.018	0.545	0.803	0.279	AMP^e^	0.937	0.089
GGGFGGGHGGGGGGGFGGGF	AMP	0.549	AMP	0.503	AMP	AMP	0.991	0.820	0.634	0.693	AMP	0.903	0.366
GEFPWMM	AMP	0.952	AMP	0.502	AMP	NAMP	0.001	0.667	0.614	0.493	Non‐AMP^e^	0.375	0.050
WWDSPLLRPF	NAMP	0.007	NAMP	0.314	AMP	NAMP	0.031	0.559	0.377	0.144	Non‐AMP	0.334	0.079
DNSFFGGF	AMP	0.993	NAMP	0.405	NAMP	NAMP	0.026	0.538	0.476	0.475	Non‐AMP^e^	0.331	0.059
FNPPGFAF	NAMP	0.007	NAMP	0.470	AMP	NAMP	0.256	0.556	0.706	0.466	AMP^e^	0.790	0.079
FGAPVFF	NAMP	0.119	AMP	0.585	AMP	NAMP	0.364	0.488	0.648	0.423	AMP^e^	0.731	0.099
SQFWFGFPQ	NAMP	0.073	NAMP	0.293	AMP	NAMP	0.023	0.593	0.216	0.285	AMP^e^	0.700	0.139
GGPFGGGPF	AMP	0.936	NAMP	0.313	AMP	NAMP	0.346	0.659	0.492	0.512	AMP^e^	0.917	0.139
SDFADFF	AMP	0.983	NAMP	0.335	NAMP	NAMP	0.005	0.599	0.500	0.417	Non‐AMP^e^	0.108	0.168
SDFFPGDF	NAMP	0.354	NAMP	0.373	NAMP	NAMP	0.002	0.541	0.443	0.401	Non‐AMP^e^	0.139	0.129
AYFGFPR	NAMP	0.037	NAMP	0.325	AMP	NAMP	0.103	0.258	0.457	0.214	AMP^e^	0.798	0.109
SVPSFGSEWFWFR	NAMP	0.043	NAMP	0.256	NAMP	NAMP	0.005	0.625	0.277	0.156	Non‐AMP	0.374	0.119
RFPIFFH	AMP	0.512	NAMP	0.487	AMP	NAMP	0.218	0.672	0.657	0.549	AMP^e^	0.869	0.198
QFPWQAF	NAMP	0.004	NAMP	0.301	AMP	NAMP	0.029	0.599	0.250	0.290	AMP^e^	0.566	0.109
AFGAPVFF	NAMP	0.380	AMP	0.591	AMP	NAMP	0.443	0.404	0.646	0.377	AMP^e^	0.722	0.119
FGFDGDFFGR	NAMP	0.000	NAMP	0.275	AMP	NAMP	0.018	0.555	0.467	0.401	AMP	0.570	0.287
KAAGGGGGIGGGIGGGIGGGIGGGIGGGIGGGL	AMP	0.972	AMP	0.733	AMP	AMP	0.999	0.857	0.658	0.667	AMP	0.999	0.505
FSWDWGPA	NAMP	0.003	NAMP	0.357	AMP	NAMP	0.131	0.713	0.576	0.237	AMP^e^	0.848	0.059
GDGGLGGGIGGGHGGGIGGGIGGGIGGGIGGGH	AMP	0.985	AMP	0.654	AMP	AMP	0.998	0.888	0.700	0.735	AMP	0.997	0.455
AYFGFPRRFLLPK	AMP	0.682	AMP	0.744	AMP	NAMP	0.374	0.468	0.668	0.541	AMP	0.946	0.564
AFWSGPL	NAMP	0.008	NAMP	0.316	AMP	NAMP	0.130	0.309	0.177	0.088	AMP^e^	0.917	0.020
FIDNIFRF	NAMP	0.002	NAMP	0.362	AMP	NAMP	0.143	0.543	0.617	0.437	AMP^e^	0.548	0.267
AAPMFDMM	AMP	0.985	AMP	0.620	NAMP	NAMP	0.001	0.644	0.559	0.449	Non‐AMP^e^	0.258	0.109
QAGAWLPF	NAMP	0.002	NAMP	0.404	AMP	NAMP	0.057	0.208	0.192	0.084	AMP^e^	0.717	0.059
DKMPWFKGW	AMP	0.62	AMP	0.507	AMP	NAMP	0.439	0.730	0.774	0.305	AMP^e^	0.832	0.129
ADQLFRMF	NAMP	0.024	AMP	0.569	AMP	NAMP	0.012	0.517	0.345	0.370	Non‐AMP^e^	0.350	0.089
FSNPIFRIF	NAMP	0.008	NAMP	0.463	AMP	AMP	0.525	0.587	0.831	0.567	AMP^e^	0.839	0.276
GPFMPGFS	AMP	0.961	AMP	0.507	AMP	NAMP	0.145	0.734	0.536	0.725	AMP^e^	0.747	0.040
FQPSFLGM	NAMP	0.154	NAMP	0.430	AMP	NAMP	0.049	0.777	0.345	0.742	AMP^e^	0.732	0.069
GWPLDKF	NAMP	0.013	NAMP	0.350	AMP	NAMP	0.015	0.353	0.579	0.091	AMP^e^	0.793	0.109
LGSGWPF	NAMP	0.096	NAMP	0.321	AMP	NAMP	0.150	0.423	0.160	0.138	AMP^e^	0.783	0.050
SFQEFPPLGRF	NAMP	0.014	NAMP	0.090	NAMP	NAMP	0.006	0.529	0.330	0.468	Non‐AMP	0.154	0.109
GGFGGDLGGGHGGFSGGF	NAMP	0.245	NAMP	0.356	AMP	AMP	0.913	0.796	0.609	0.642	AMP	0.587	0.317
AIWMGPL	NAMP	0.002	NAMP	0.337	AMP	NAMP	0.231	0.646	0.705	0.342	AMP^e^	0.725	0.208
FIADHPFIF	NAMP	0.185	NAMP	0.454	AMP	NAMP	0.095	0.595	0.580	0.447	Non‐AMP^e^	0.122	0.139
WGPEWGL	NAMP	0.010	NAMP	0.350	AMP	NAMP	0.123	0.787	0.785	0.409	Non‐AMP^e^	0.309	0.208
SDRFGFL	NAMP	0.054	NAMP	0.337	NAMP	NAMP	0.022	0.284	0.240	0.242	AMP^e^	0.669	0.030
PQGPFAF	AMP	0.984	NAMP	0.436	AMP	NAMP	0.134	0.500	0.438	0.456	AMP^e^	0.671	0.030
GGGGGFGGGF	AMP	0.702	NAMP	0.293	AMP	NAMP	0.312	0.754	0.582	0.593	AMP	0.886	0.446
GGGFGGGF	AMP	0.739	NAMP	0.272	AMP	NAMP	0.208	0.717	0.574	0.558	AMP^e^	0.897	0.416
KESQFWFGFPQRF	NAMP	0.084	NAMP	0.219	AMP	NAMP	0.024	0.635	0.362	0.330	Non‐AMP	0.490	0.198
QFSYLPW	NAMP	0.000	NAMP	0.281	NAMP	NAMP	0.002	0.184	0.096	0.078	AMP^e^	0.620	0.069
GSGWGWLGY	NAMP	0.002	NAMP	0.379	AMP	NAMP	0.236	0.710	0.444	0.500	AMP^e^	0.957	0.168
QIPRLLGPGLNKAGKFPGLL	AMP	0.818	AMP	0.951	AMP	AMP	0.987	0.735	0.283	0.500	AMP	0.998	0.485
GEFPWMIG	NAMP	0.109	NAMP	0.459	AMP	NAMP	0.003	0.649	0.667	0.317	Non‐AMP^e^	0.134	0.119
ADMKHWPF	NAMP	0.061	NAMP	0.474	NAMP	NAMP	0.004	0.625	0.596	0.418	Non‐AMP^e^	0.069	0.168
EAYFGFPRRFLLPK	NAMP	0.278	NAMP	0.362	AMP	NAMP	0.031	0.270	0.594	0.287	AMP	0.613	0.376
ANWGPEWGLL	NAMP	0.005	NAMP	0.300	AMP	NAMP	0.260	0.648	0.807	0.130	Non‐AMP	0.348	0.119
QEFPPLGRF	NAMP	0.015	NAMP	0.260	AMP	NAMP	0.006	0.500	0.232	0.369	Non‐AMP^e^	0.325	0.059
FLWGPAL	NAMP	0.002	NAMP	0.395	AMP	AMP	0.672	0.259	0.511	0.076	AMP^e^	0.856	0.188
VPFPRLHFFMPGFAPL	AMP	0.757	AMP	0.518	AMP	NAMP	0.305	0.659	0.784	0.701	AMP	0.989	0.337
GGGGGLGGGGGLGGGGGLGGGK	NAMP	0.010	AMP	0.522	AMP	AMP	0.658	0.891	0.598	0.782	AMP	0.800	0.396
SDKMPWFKGW	NAMP	0.432	NAMP	0.390	NAMP	NAMP	0.301	0.715	0.634	0.274	AMP	0.715	0.218
SAPIWQF	NAMP	0.039	NAMP	0.208	AMP	NAMP	0.021	0.251	0.215	0.130	Non‐AMP^e^	0.343	0.129
YSPYFPL	AMP	0.970	NAMP	0.293	NAMP	NAMP	0.093	0.371	0.286	0.173	AMP^e^	0.695	0.119
GWAEPIRFL	NAMP	0.025	NAMP	0.299	AMP	NAMP	0.031	0.061	0.797	0.031	Non‐AMP^e^	0.400	0.208
SGPFGQIF	NAMP	0.039	NAMP	0.216	AMP	NAMP	0.084	0.639	0.363	0.471	AMP^e^	0.537	0.059
GGGGGLGGGGGL	NAMP	0.008	NAMP	0.397	AMP	NAMP	0.160	0.892	0.585	0.717	AMP	0.651	0.525
SSPDLNFF	AMP	0.999	AMP	0.555	NAMP	NAMP	0.017	0.361	0.392	0.446	Non‐AMP^e^	0.422	0.069
LPDQWDWRL	NAMP	0.052	NAMP	0.249	NAMP	NAMP	0.004	0.500	0.568	0.237	Non‐AMP^e^	0.025	0.030
GQFPWMVAL	NAMP	0.038	NAMP	0.380	AMP	NAMP	0.014	0.265	0.316	0.122	AMP^e^	0.627	0.040
QAAFPAPF	NAMP	0.256	NAMP	0.481	AMP	NAMP	0.094	0.558	0.555	0.500	AMP^e^	0.721	0.030
SDKDKFFYF	AMP	0.531	NAMP	0.395	NAMP	NAMP	0.051	0.500	0.476	0.383	Non‐AMP^e^	0.287	0.238
FAFFDNL	NAMP	0.150	NAMP	0.312	NAMP	NAMP	0.009	0.528	0.494	0.406	Non‐AMP^e^	0.303	0.099
GEWYPDHHFRLL	NAMP	0.023	NAMP	0.064	NAMP	NAMP	0.002	0.411	0.537	0.173	Non‐AMP	0.352	0.178
SNPPLWPY	AMP	0.924	NAMP	0.451	NAMP	NAMP	0.051	0.633	0.181	0.294	AMP^e^	0.613	0.089
ADKGFLW	NAMP	0.000	NAMP	0.327	AMP	NAMP	0.020	0.500	0.740	0.147	Non‐AMP^e^	0.468	0.238
GGGYGGGLGGGF	NAMP	0.007	NAMP	0.373	AMP	AMP	0.726	0.875	0.599	0.773	AMP	0.900	0.188
SFSNPIFRIF	NAMP	0.007	NAMP	0.342	AMP	NAMP	0.145	0.618	0.800	0.606	AMP	0.895	0.287
KDWLDSPWSGF	NAMP	0.021	NAMP	0.125	NAMP	NAMP	0.012	0.521	0.470	0.123	AMP	0.509	0.089
SAGGLIGAGGLIGTGGLIGAR	NAMP	0.334	AMP	0.700	AMP	AMP	0.933	0.902	0.667	0.576	AMP	0.825	0.149
FMPGFAPL	AMP	0.535	AMP	0.546	AMP	NAMP	0.185	0.664	0.700	0.725	AMP^e^	0.942	0.158
IADHPFIF	NAMP	0.249	NAMP	0.449	AMP	NAMP	0.085	0.646	0.426	0.431	Non‐AMP^e^	0.144	0.109
DARPLPEWFDKAKIGIF	NAMP	0.057	NAMP	0.111	NAMP	NAMP	0.052	0.122	0.525	0.077	Non‐AMP	0.057	0.050
FGGPEMW	AMP	0.789	NAMP	0.470	AMP	NAMP	0.003	0.665	0.582	0.393	Non‐AMP^e^	0.139	0.059
PLPQGPF	NAMP	0.417	NAMP	0.335	AMP	NAMP	0.056	0.450	0.242	0.310	AMP^e^	0.786	0.040
WFNPSIL	NAMP	0.000	NAMP	0.337	AMP	NAMP	0.163	0.461	0.578	0.180	AMP^e^	0.681	0.050
SNPIFRIF	NAMP	0.055	NAMP	0.383	AMP	AMP	0.520	0.617	0.795	0.667	AMP^e^	0.576	0.168
SSLFSPW	NAMP	0.121	NAMP	0.186	NAMP	NAMP	0.023	0.590	0.193	0.281	AMP^e^	0.824	0.168
ASDKMPWFKGW	AMP	0.546	NAMP	0.374	AMP	NAMP	0.250	0.697	0.598	0.249	AMP	0.606	0.119
PDQWDWRLY	NAMP	0.153	NAMP	0.306	NAMP	NAMP	0.001	0.570	0.418	0.307	Non‐AMP^e^	0.161	0.030
GPAPLQPLPKL	NAMP	0.019	NAMP	0.247	AMP	NAMP	0.343	0.103	0.158	0.054	AMP	0.897	0.228
PSPPGGPL	NAMP	0.419	NAMP	0.423	AMP	NAMP	0.031	0.319	0.169	0.354	AMP^e^	0.778	0.376
AVPIPPRFG	AMP	0.688	NAMP	0.453	AMP	NAMP	0.470	0.459	0.362	0.679	AMP^e^	0.745	0.119
GWDDPVLADPLKRKIPLRRF	AMP	0.576	NAMP	0.279	AMP	AMP	0.896	0.100	0.164	0.048	Non‐AMP	0.014	0.386
GGGFGGGAGGGF	AMP	0.659	NAMP	0.419	AMP	AMP	0.984	0.834	0.632	0.696	AMP	0.722	0.347
DKMPWFK	AMP	1.000	AMP	0.566	AMP	NAMP	0.237	0.715	0.663	0.490	AMP^e^	0.615	0.228
SGSFLSPW	NAMP	0.017	NAMP	0.174	AMP	NAMP	0.038	0.528	0.095	0.184	AMP^e^	0.923	0.089
NADHPFIF	NAMP	0.008	NAMP	0.322	AMP	NAMP	0.007	0.471	0.414	0.386	Non‐AMP^e^	0.145	0.059
WTGIGFM	NAMP	0.004	NAMP	0.330	AMP	NAMP	0.035	0.651	0.661	0.381	AMP^e^	0.861	0.317
AQFGFDGDFFGR	NAMP	0.017	NAMP	0.204	NAMP	NAMP	0.016	0.394	0.277	0.261	Non‐AMP	0.183	0.228
APVWAPGL	NAMP	0.005	NAMP	0.338	AMP	NAMP	0.198	0.230	0.370	0.100	AMP^**e**^	0.785	0.168
PLPQGPFAF	AMP	0.540	NAMP	0.417	AMP	NAMP	0.104	0.420	0.400	0.420	AMP^**e**^	0.789	0.079

The antimicrobial activity, general and specific to bacteria, viruses and fungi, was evaluated using different tools.

^a^ CAMPR3 (http://www.camp3.bicnirrh.res.in/).

^b^ iAMPpred (http://cabgrid.res.in:8080/amppred/).

^c^ Antimicrobial Peptide Scanner vr.2 (https://www.dveltri.com/ascan/v2/ascan.html).

^d^ Macrel (https://big‐data‐biology.org/software/macrel). Each tool might have a different threshold, but higher score indicated the greater the likelihood of the peptide being bioactive. All these tools were accessed in February 2024.

In relation to the prediction of antimicrobial activity of the peptides, several tools were employed. CAMPR3 has been reported to be the most efficient tool for this purpose, compared to several other tools, according to Gabere and Noble.^33^ Similarly, Ruiz‐Blanco *et al*.,^34^ indicated the high potential and accuracy of iAMPred, Antimicrobial Peptide Scanner vr.2 as well as AMPDiscover and ABP‐Finder as AMP prediction tools. For this reason, these tools (CAMPR3, and Antimicrobial Peptide Scanner vr.2) were chosen, together with Macrel (a tool including a set of 22 peptide features), as a tool not evaluated in those reports comparing tools. Taking into consideration the values obtained in the different tools, the most promising sequences which could be antimicrobial are FGPKGVGFGMGAGALTMA, GGGGGIGGGIGGGIGGGIGGGIGGGIGGGL, GGGFGGGHGGGGGGGFGGGF, KAAGGGGGIGGGIGGGIGGGIGGGIGGGIGGGL, GDGGLGGGIGGGHGGGIGGGIGGGIGGGIGGGH and QIPRLLGPGLNKAGKFPGLL. These are the only peptides among all the evaluated sequences which were predicted as AMPs according to the CAMPR3 tool, considering the different models used by that tool. According to the authors, CAMPR3 contains 10 247 AMP sequences, of which 4857 are experimentally validated and 5390 are predicted, together with 757 antimicrobial structures. In the case of iAMPpred, the prediction is based on amino acid compositions, structural features (*α*‐helix, *β*‐sheet and turn structure propensity), and physicochemical properties (isoelectric point, hydrophobicity and net charge). Considering these results, and how the different tools take into consideration different and novel peptide features, with different algorithms, the purpose of this study was to correlate all the information by using the predicted bioactivity in the *T*. *molitor* hydrolysate peptidome.

In relation to peptides with the highest activity, a glycine‐rich antibacterial peptide directed against Gram‐positive bacteria has also been reported in the woodlouse *Armadillidium vulgare*.^35^ For instance, gloverins are 8–30 kDa glycine‐rich AMPs that were originally identified from the haemolymph of gigantic silk moth pupae. In aqueous solution, gloverins have a flexible random‐coil structure. Different insect gloverins are active against viruses, bacteria and fungi.^36^ On top of that, it has been described that cysteine residues in some AMPs are often connected by disulfide bridges, making them highly stable.^20^ Regarding AMPs, the relevance of the C, G, K, R and S amino acids has been highlighted. In addition, the residues would ideally be positioned in the N‐terminal, especially for G, K, R and A. Similarly, residues N, S, C and G prefer the C‐terminal.^37^ Indeed, the peptides with highest PeptideRanker score in TMH10A are sequences right in G, and 35 sequences (from both TMF and TMH10A) contain a G in the N‐terminal and 8 in the C‐terminal.

It has been proposed that research should focus on digesting proteins with proteases that release sequences with positively charged amino acids at their terminal, like trypsin, based on the molecular characteristics that these peptides often exhibit (primarily cationic and amphiphilic *α*‐helical peptide molecules).^11^ However, in this study, it was observed how the use of Alcalase‐hydrolysed proteins might also be an adequate strategy to improve the antimicrobial activity of peptides, as this enzyme acts towards hydrophobic residues, which have also been shown to be effective antimicrobial agents.^38^ Similarly to the identified peptide AGDKKIKIGINGFGRIGRL^39^ proved to inhibit *Escherichia coli* and *Staphylococcus aureus* with a minimal inhibitory concentration of 0.064 and 0.512 mg mL^−1^, respectively, 18 of the sequences identified had the motif AG at the terminal, such as AGYLRPW, which showed adequate scores in the prediction tools, or AGPGHWNDPDML. Terminal RL was also found in some of the peptides identified in the samples, such as LPDQWDWRL or NWFIDRL, also with medium/high values of antimicrobial activity in the different tools. Similarly, the motif PLL identified in the antimicrobial peptide WWDSPLLRPF was found in one of the peptides in the hydrolysate. The motif GD (from the antibacterial peptide GDVIAIR), reported by Aguilar‐Toalá *et al*.,^40^ was found as well in 11 of the identified sequences, including one of the peptides proposed to be highly bioactive according to the tools (GDGGLGGGIGGGHGGGIGGGIGGGIGGGIGGGH). Following a similar approach, the VLA residue of the antifungal peptide VLALHSVPK identified by Arulrajah *et al*.,^41^ was found in the peptide GWDDPVLADPLKRKIPLRRF.

In the evaluation of complete peptidomes, an interesting approach is trying to encompass all the information available, in order to be able to predict or understand the underlying mechanisms by which specific sequences could be correlated to some bioactivities. For this reason, further *in silico* analyses, including structure–activity relationship and molecular docking, were performed.

#### Structure–activity relationship

**Figure 1:**
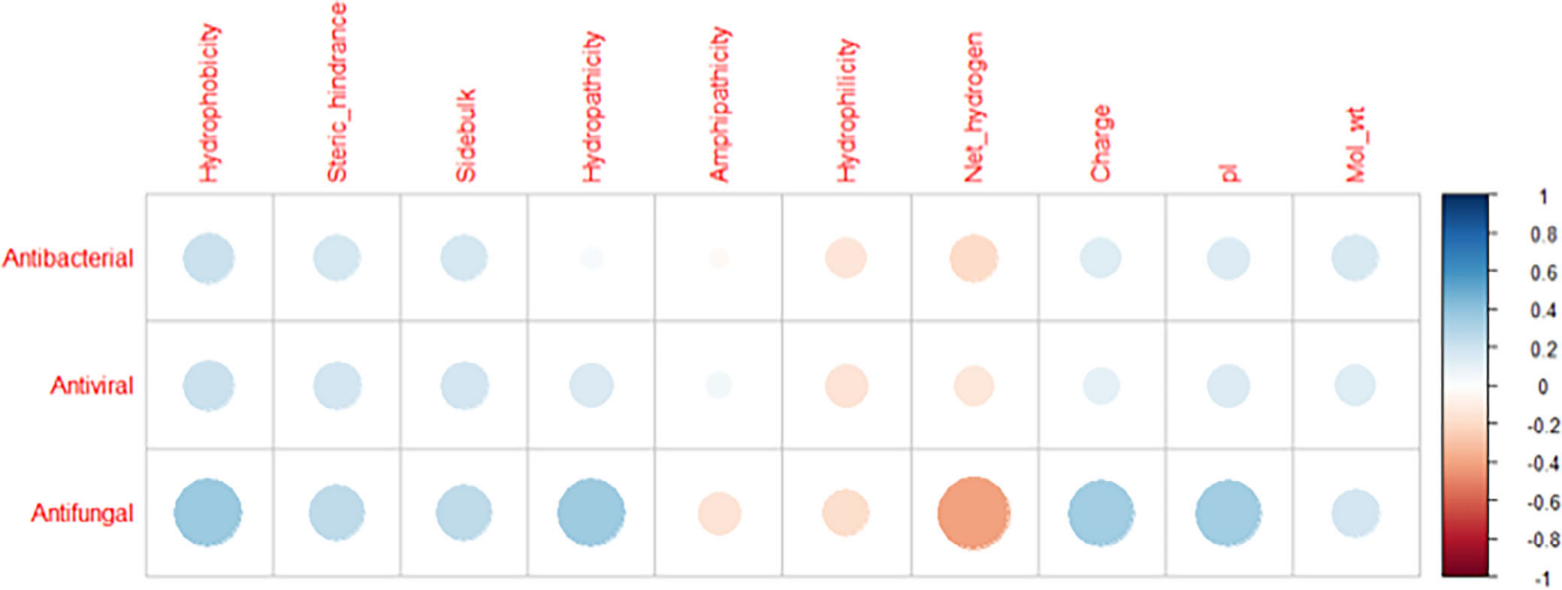
Correlation heatmap between peptide characteristics (columns) and their antifungal, antiviral and antibacterial capacities (rows). The colour and size of each circle represent Pearson's correlation coefficient.

The connections between the physicochemical properties as predicted by ToxinPred and the scores obtained from the specific antimicrobial tools (for bacteria, fungi and viruses) were investigated and the results depicted in Fig. 1. Both size and colour intensity show the level of correlation, which is why the most coloured ones are the largest. Strong positive correlations, meaning values higher than 0.5, between the bioactivities and any of the parameters evaluated were not found. However, a correlation value between 0.3 and 0.5 might be considered moderate, and in this range, the correlation between antifungal activity and hydrophobicity (0.368), hydropathicity (0.359), net hydrogen (−0.407), charge (0.344) and pI (0.345) was found (Table S1). In the case of antibacterial and antiviral peptides, only a low correlation was shown with the parameters. Through strong interactions with the negatively charged phospholipid bilayer of the microbial membrane, positively charged peptides exert their antimicrobial activity. The structure of the amphipathic peptides then allows penetration into the hydrophobic core, causing intracellular leakage through pore formation and disruption of the cytoplasmic membrane, ultimately leading to cell lysis.^42^ The results obtained in the correlation maps support the literature in relation to the antifungal activity of peptides, in this case, specifically for insect peptides. In fact, amphipathic peptides, which have hydrophobic and hydrophilic regions, are often effective at disrupting microbial membranes.

Net hydrogen of peptides is related to their capacity to act as hydrogen donors.^43^ It is interesting that no correlation was found in relation to the molecular weight of the peptides. The relationship between molecular weight and antimicrobial activity can vary depending on the specific target microorganism. Some peptides may be more effective against certain types of bacteria or fungi. It has been described that smaller peptides may have better penetration ability across bacterial membranes, allowing them to reach their target sites more efficiently, while at the same time the AMPs are cationic (positive charge). However, the balance between size and charge is critical; peptides that are too small may lack the necessary interactions with microbial membranes, while peptides that are too large may have reduced membrane penetration.

The side bulk of amino acids of AMPs has a significant impact upon their interactions with membranes and consequently their antimicrobial bioactivities. The amphiphilicity and specific bioactivities of short peptide amphiphiles can be strongly impacted by modifications inside chain length and branching. Modification of side‐chain residues can influence side‐chain properties such as cationicity, hydrophobicity, steric factors, conformational stability and hydrogen bonding. This can reveal important information about the structure–activity relationships and mechanism of action of AMPs. Furthermore, it has been observed that the hydrophobicity of the side‐chain of AMPs and their ability to combat certain bacteria are positively correlated. Therefore, bioactivities, interactions with membranes and selectivity toward distinct bacteria of AMPs are all significantly influenced by their side bulk.^44^ The side bulk is related to peptides' conformation and stability, ultimately affecting bioactivity.^45^

In relation to the net hydrogen of peptides, which appears to be the most influencing factor, the following aspects must be taken into consideration. In relation to hydrophobic interactions, numerous AMPs interact with the lipid membranes of bacteria through hydrophobic areas. In order for a peptide to penetrate the microbial membrane, damage its integrity and cause cell death, these hydrophobic interactions are necessary. Hydrogen atoms in these hydrophobic areas can change the overall hydrophobicity of the peptide, which can change how well it interacts with and breaks down microbial membranes.^46^ In addition, although not evaluated in this study, a peptide's secondary structure is essential to its antimicrobial action. These secondary structures are largely stabilized by hydrogen bonding. The overall stability and shape of a peptide can be impacted by hydrogen bonding, which can also have an impact on how the peptide interacts with other biological components or microbial membranes.^47^

**Figure 2:**
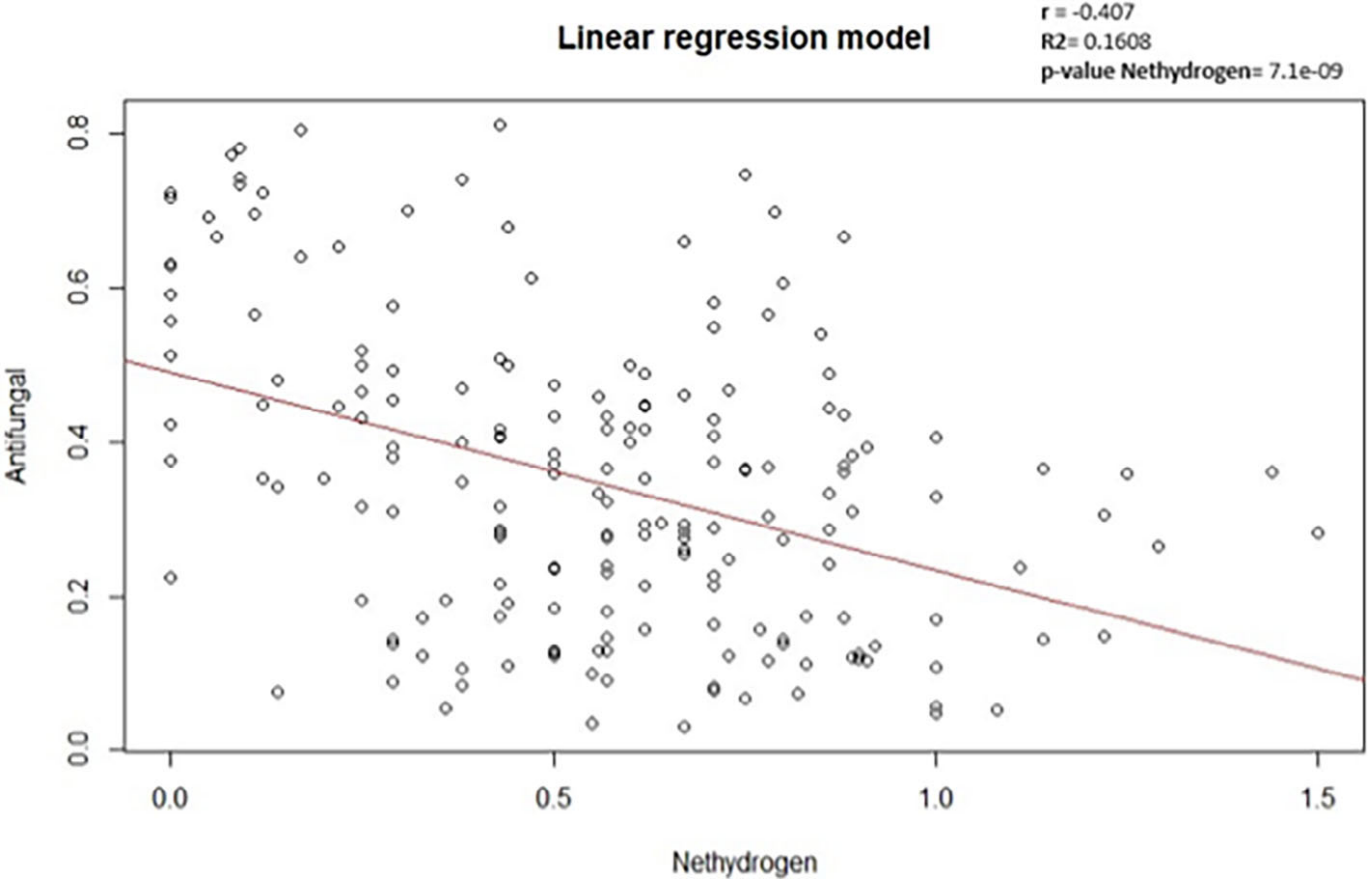
Linear regression model. The *x*‐axis represents the levels of net hydrogen as an independent variable. The *y*‐axis represents the antifungal capacity as the dependent variable. The red line represents the created linear regression model. The *r* value represents Pearson's correlation coefficient between the two variables. *R* ^2^ shows that the model is able to explain 16% of the variance of the data. The *P* value of net hydrogen shows that the variable is significant for the model.

One field that has been scarcely explored is the charge distribution, since the presence of hydrogen atoms in the amino acid side chains contributes to the overall charge distribution of a peptide. Many AMPs are cationic, meaning they have a net positive charge. The electrostatic interaction between the positively charged peptide and the negatively charged microbial membrane enhances the ability of the peptide to bind to and disrupt the membrane. However, as concluded by Duque *et al*.,^48^ there is no software available that allows one to discriminate charge distribution in AMPs and predict the biological effects of this parameter. On top of that, the pH sensitivity of a peptide, which can be influenced by the presence of hydrogen ions (protons), may impact its antimicrobial activity. Some AMPs show increased activity at acidic pH, which is relevant in environments such as the skin or acidic compartments within cells.^49^ It is significant to note that the intricate interactions between a number of variables that determine peptides' antimicrobial activity include their general structure, their particular amino acid sequence and other physicochemical characteristics. Then, after assessing the strongest correlations between antifungal, antiviral and antibacterial capacities and the different peptides, the strongest Pearson's correlation was acquired between antifungal capacity and net hydrogen (*r* = −0.407). Nonetheless, it is always necessary to keep in mind that in this type of analysis the correlations could be coincidental, and it is required to continue testing and analysing and validating the data. To check that the relationship established between the two variables was not coincidental, the behaviour was modelled by simple linear regression to see if net hydrogen levels could be explaining the antifungal capacity of the peptides. The results showed that net hydrogen had a significant effect (*P* < 0.05) on antifungal capacity. However, regression analysis (Fig. 2) indicated that this variable only managed to explain 16% of the variance in the antifungal capacity data (*R* ^2^ = 0.1608), which leads us to believe that there are many more factors and variables influencing the antifungal capacity of the peptides.

Although these results show evidence suggesting a correlation between the physicochemical properties of peptides and their antimicrobial activities, the findings are not entirely conclusive. The data indicate that certain molecular features may influence antibacterial, antiviral and antifungal effects, but these relationships are complex and not uniformly predictive across all peptides examined. Further research is necessary to elucidate the connections and to determine the precise mechanisms involved. Future studies with larger datasets and more diverse peptide structures are needed to validate these preliminary observations.

#### Molecular docking

On top of the description of the antimicrobial potential of the identified peptidomes in the previous section, molecular docking of sequences was done with different receptors, according to the results obtained.

##### Bacterial receptors

**Figure 3:**
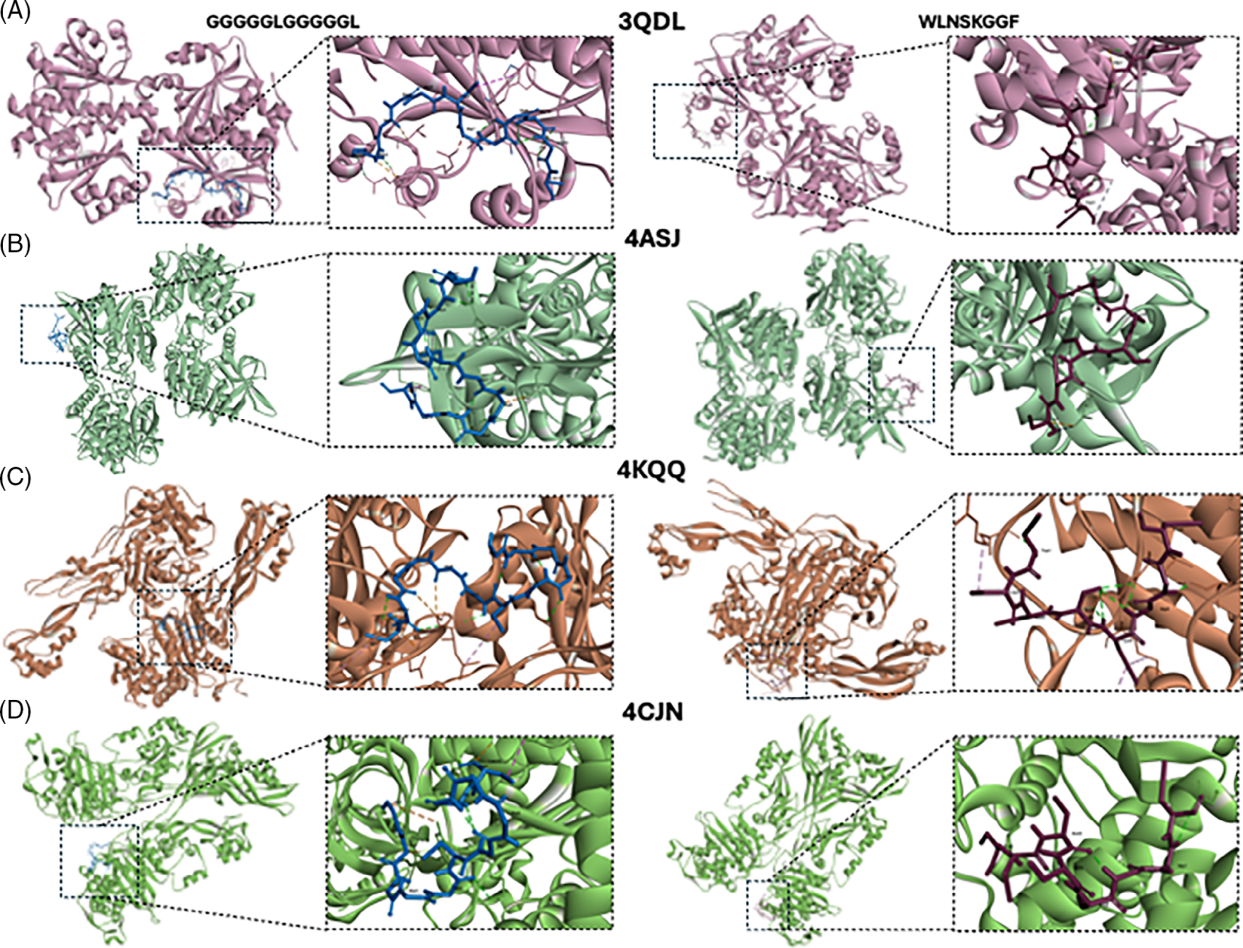
Visualization of the structures of receptor–peptide complexes using Biovia Discovery Studio Visualizer. (A) RdxA–GGGGGLGGGGGL/WLNSKGGF binding site and interactions (PDB: 3QDL); (B) RmIA–GGGGGLGGGGGL/WLNSKGGF binding site and interactions (PDB: 4ASJ); (C) PBP2A–GGGGGLGGGGGL/WLNSKGGF binding site and interactions (PDB: 4CJN); and (D) PBP3–GGGGGLGGGGGL/WLNSKGGF binding site and interactions (PDB: 4KQQ).

**Figure 4:**
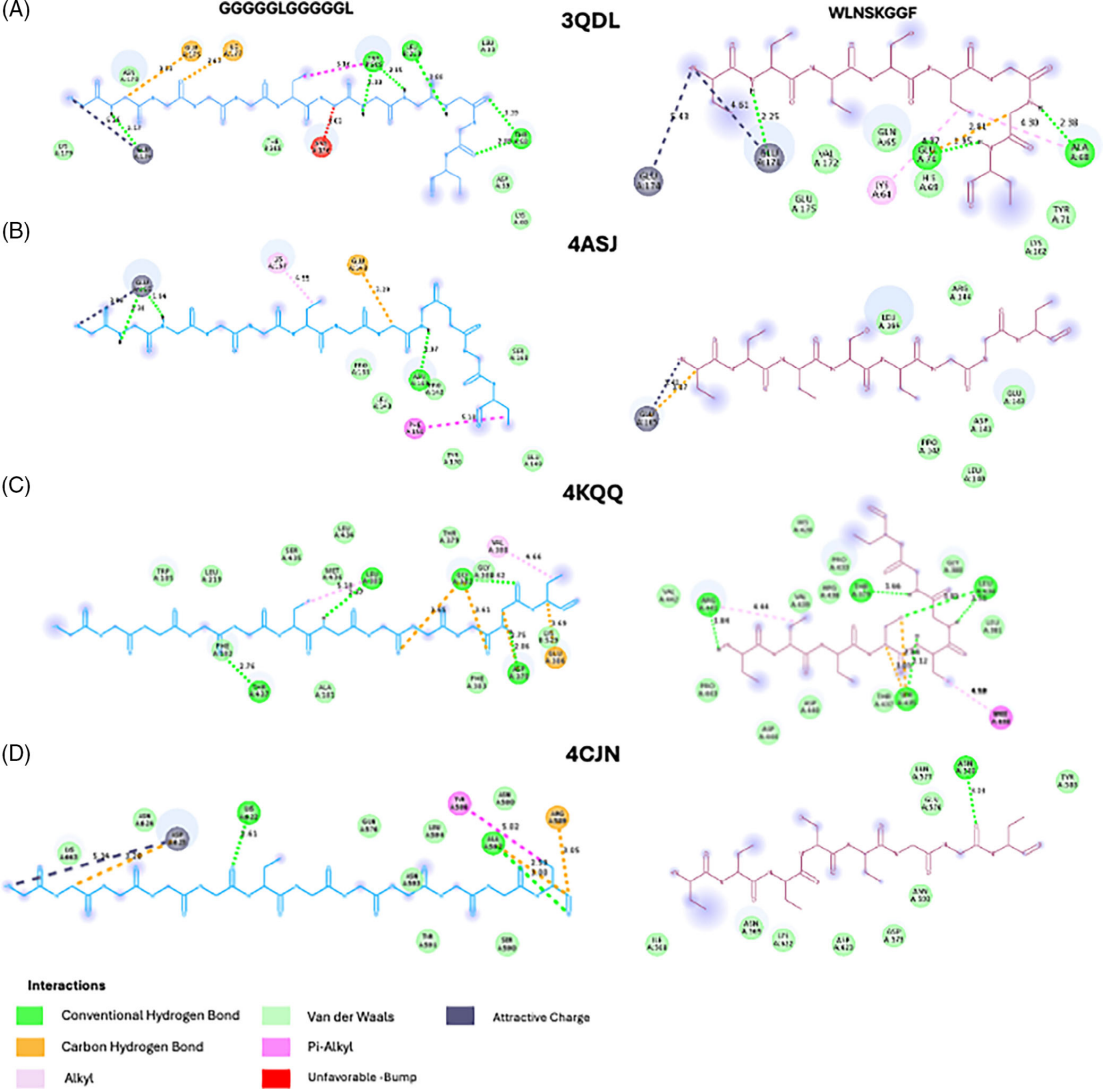
Analysis of 2D residue diagrams for selected peptides with their respective interaction bonds with bacterial receptors: (A) oxygen‐insensitive NADPH nitroreductase of *Helicobacter pylori* (PDB: 3QDL); (B) glucose‐1‐phosphate thymidylyltransferase (RmIA) from *Pseudomonas aeruginosa* (PDB: 4ASJ); (C) penicillin‐binding protein 2a from *Staphylococcus aureus* subsp. *aureus* Mu50 (PDB: 4CJN); and (D) penicillin‐binding protein 3 from *Pseudomonas aeruginosa* (PDB: 4KQQ). These were visualized using Biovia Discovery Studio Visualizer.

Although the three peptides proposed as highly antibacterial, according to the score, were GDGGLGGGIGGGHGGGIGGGIGGGIGGGIGGGH, GGGGGLGGGGGL and SAGGLIGAGGLIGTGGLIGAR (scores higher than 0.888), those having a high molecular weight (first sequence having 33 residues and the third one having 21) could not be subjected to molecular docking analysis due to the limitations of the tool. For this reason, the peptides subjected to the analyses were GGGGGLGGGGGL and WLNSKGGF, the latter having a score of 0.872, which is not very different from the threshold of the peptides mentioned previously. These peptides were analysed in relation to their interaction with the three different virulent bacterial strains. In Figs 3 and 4, the visualization of the interaction of each peptide with the different bacterial receptors analysed is depicted, together with the results presented in Table 3.

**Table 3:** Molecular docking results for receptor–peptide binding including the affinity of each peptide with the target receptors, the cluster size and the interaction (position, type and distance)

	Receptor	Peptide	Docking results				
			Possible best result				
			Affinity (kcal mol^−1^)	Cluster size	Interactions		
					Interaction residues	Bond distance (Å)	Type of interaction bond
Bacteria	3QDL	GGGGGLGGGGGL	−12.5	48	Gly1‐Glu174 (A)	5.25	*Attractive Charge*
					Gly2‐Glu174 (B)	2.17	*Conventional Hydrogen*
					Gly2‐Glu175 (A)	3.23	*Carbon Hydrogen*
					Gly3‐Ile177 (A)	2.63	*Carbon Hydrogen*
					Leu6‐Trp209 (B)	5.16	*Pi‐Alkyl*
					Gly7‐Arg176 (A)	2.02	*Unfavourable Bump*
					Gly8‐Tr209 (B)	2.00	*Conventional Hydrogen*
					Gly9‐Trp209 (B)	2.15	*Conventional Hydrogen*
					Gly10‐Leu210 (B)	2.88	*Conventional Hydrogen*
					Gly10‐Thr58 (A)	3.39	*Conventional Hydrogen*
					Gly11‐Thr58 (A)	2.89	*Conventional Hydrogen*
		WLNSKGGF	−13.5	49	Trp1‐Glu174 (A)	5.43	*Attractive Charge*
					Trp1‐Glu171 (A)	4.61	*Attractive Charge*
					Leu2‐Glu171 (A)	2.25	*Conventional Hydrogen*
					Lys5‐Lys64 (A)	4.37	*Alkyl*
					Lys5‐Ala68 (A)	4.30	*Alkyl*
					Gly7‐Ala68 (A)	2.38	*Conventional Hydrogen*
					Gly7‐Glu74 (A)	2.81	*Carbon Hydrogen*
					Phe8‐Glu74 (A)	1.95	*Conventional Hydrogen*
	4ASJ	GGGGGLGGGGGL	−13.0	62	Gly1‐Glu165 (A)	3.82	*Attractive Charge*
					Gly2‐Glu165 (A)	2.30	*Conventional Hydrogen*
					Gly3‐Glu165 (A)	1.94	*Conventional Hydrogen*
					Leu6‐Lys167 (A)	4.55	*Alkyl*
					Gly8‐Glu143 (A)	3.29	*Carbon Hydrogen*
					Gly9‐Asn169 (A)	2.07	*Conventional Hydrogen*
					Leu12‐Phe150 (A)	5.18	*Pi‐Alkyl*
		WLNSKGGF	−14.1	29	Trp1‐Glu165 (A)	3.41	*Attractive Charge*
						3.07	*Carbon Hydrogen*
	4CJN	GGGGGLGGGGGL	−10.1	59	Gly1‐Asp623 (A)	5.34	*Attractive Charge*
					Gly2‐Asp623 (A)	3.20	*Carbon Hydrogen*
					Gly5‐Lys622 (A)	2.61	*Conventional Hydrogen*
					Leu12‐Tyr588 (A)	5.02	*Pi‐Alkyl*
					Leu12‐Ala592	2.59	*Carbon Hydrogen*
						3.00	*Conventional Hydrogen*
					Leu12‐Arg589 (A)	3.05	*Carbon Hydrogen*
		WLNSKGGF	−13.4	21	Gly7‐As580 (A)	3.03	*Conventional Hydrogen*
	4KQQ	GGGGGLGGGGGL	−13.3	40	Gly4‐Thr437 (A)	2.76	*Conventional Hydrogen*
					Leu6‐Leu381 (A)	5.18	*Alkyl*
					Gly7‐Leu381 (A)	2.37	*Conventional Hydrogen*
					Gly8‐Gly382 (A)	3.65	*Carbon Hydrogen*
					Gly10‐Glu382 (A)	3.61	*Carbon Hydrogen*
					Gly11‐Asp378 (A)	2.75	*Carbon Hydrogen*
						2.86	*Conventional Hydrogen*
					Gly11‐Gly380 (A)	2.62	*Conventional Hydrogen*
					Leu12‐Glu386 (A)	3.69	*Carbon Hydrogen*
					Leu12‐Val388 (A)	4.66	*Alkyl*
		WLNSKGGF	−15.5	10	Trp1‐Arg441 (A)	1.84	*Conventional Hydrogen*
					Leu2‐Arg441 (A)	4.44	*Alkyl*
					Ser4‐Ser435 (A)	3.05	*Carbon Hydrogen*
						2.84	*Carbon Hydrogen*
						2.12	*Conventional Hydrogen*
					Ser4‐Leu434 (A)	3.05	*Conventional Hydrogen*
					Lys5‐Met436 (A)	4.10	*Alkyl*
					Lys5‐Phe182 (A)	4.92	*Pi‐Alkyl*
					Gly6‐Leu434 (A)	2.31	*Conventional Hydrogen*
					Gly7‐Thr379 (A)	1.66	*Conventional Hydrogen*
Viruses	7C2Q	GFIPYEPFLKKMMA	−14.5	20	Gly1‐Asp33 (B)	3.10	*Salt Bridge*
					Tyr5‐Glu178 (B)	2.45	*Conventional Hydrogen*
					Glu6‐Glu178 (B)	2.04	*Conventional Hydrogen*
					Lys11‐Pro184 (B)	4.77	*Alkyl*
		FSNPIFRIF	−11.0	31	Phe1‐Tyr101 (B)	3.73	*Pi‐Cation*
						5.37	*Amide Pi‐Stacked*
					Phe1‐Lys102 (B)	2.67	*Carbon Hydrogen*
					Phe1‐Phe103 (B)	2.89	*Conventional Hydrogen*
					Ser2‐Glu178 (B)	2.73	*Conventional Hydrogen*
					Asn3‐Phe103 (B)	1.94	*Conventional Hydrogen*
					Ile5‐Arg105 (B)	3.90	*Alkyl*
					Ile8‐Arg105 (B)	3.10	*Conventional Hydrogen*
					Ile8‐Phe181 (B)	2.44	*Unfavourable Acceptor‐Acceptor*
Fungi	4LXJ	GFIPYEPFLKKMMA	−10.0	23	Pro7‐Pro244 (A)	5.18	*Alkyl*
					Leu9‐Ala127 (A)	3.90	*Alkyl*
					Leu9‐His128 (A)	4.80	*Pi‐Alkyl*
					Lys10‐Ala124 (A)	5.39	*Alkyl*
					Met12‐Pro244 (A)	4.81	*Alkyl*
		FGPKGVGFGMGAGALTMA	−11.4	29	Gly9‐Asn245 (A)	2.07	*Conventional Hydrogen*
					Gly9‐Pro244 (A)	2.93	*Carbon Hydrogen*
					Met10‐Pro244 (A)	5.13	*Alkyl*
					Ala12‐Lys386	3.01	*Conventional Hydrogen*
					Gly13‐Ala124 (A)	2.64	*Carbon Hydrogen*
					Leu15‐Arg98 (A)	2.21	*Unfavourable Donor‐Donor*
						4.34	*Alkyl*
						2.95	*Conventional Hydrogen*
					Thr16‐Trp65 (A)	2.92	*Conventional Hydrogen*
					Met17‐Phe243 (A)	4.65	*Pi‐Alkyl*
					Met17‐Trp65 (A)	5.24	*Pi‐Alkyl*

The affinity values found for both peptides, interacting with the different receptors, ranged from −15.5 to −10.1 kcal mol^−1^, and it must be highlighted that the peptide WLNSKGGF had always lower energy that the other peptide, indicating that it could potentially be of higher interest to be used as a therapeutic antimicrobial agent. It must be noted that around 43% of the interactions between amino acids of the peptides and the receptors were conventional hydrogen bonds, which will most likely stabilize the interaction,^50^ potentially leading to a modification of the spatial conformation.

The receptors were obtained from different microorganisms, including *Helicobacter pylori*, *Pseudomonas aeruginosa* and *Staphylococcus aureus* subsp. *aureus* Mu50. *Helicobacter pylori* is a type of bacterium that affects the inner layer of the stomach and can lead to a number of gastrointestinal disorders. Although many people may not show any symptoms, it is believed that over half of the world's population may be infected with this bacterium, and the main strategy for eliminating it currently is antibiotic treatment. In this case, the receptor was oxygen‐insensitive NADPH nitroreductase, which is actually responsible for the susceptibility of this organism to the redox active prodrug metronidazole.^51^ One of the most known microorganisms is *Staphylococcus aureus*, which, in spite of being part of the gut microbiota, can colonize various surfaces of the human body, including the skin and mucous membranes potentially leading to several types of infections, from minor skin infections to severe, life‐threatening conditions. Penicillin‐binding protein 2a (the receptor chosen for this species) is one of the membrane‐associated enzymes that play an essential role in the peptidoglycan biosynthetic process.^52^ This same receptor was used in the case of *Pseudomonas aeruginosa*, which is an opportunistic bacterium, which can affect, for instance, people in hospital, who are immunosuppressed, or patients suffering from cystic fibrosis, affecting the bacteria directly in the lungs.^53^ In addition to that, this bacterium is highly capable of developing multidrug resistance, complicating treatment strategies.

These identified peptides in the sample could be highly contributory to antimicrobial activity towards different microorganisms, although validation is needed through experimental work, as recently reported by other authors.^14^, ^15^ Given that AMPs are cationic and contain *α*‐helices, *β*‐sheet structures and negatively charged amino acids, further studies could leverage specific proteases or microorganisms that can release these structures, investigating various reaction conditions and sources to obtain highly bioactive hydrolysates.

##### Viral receptors

**Figure 5:**
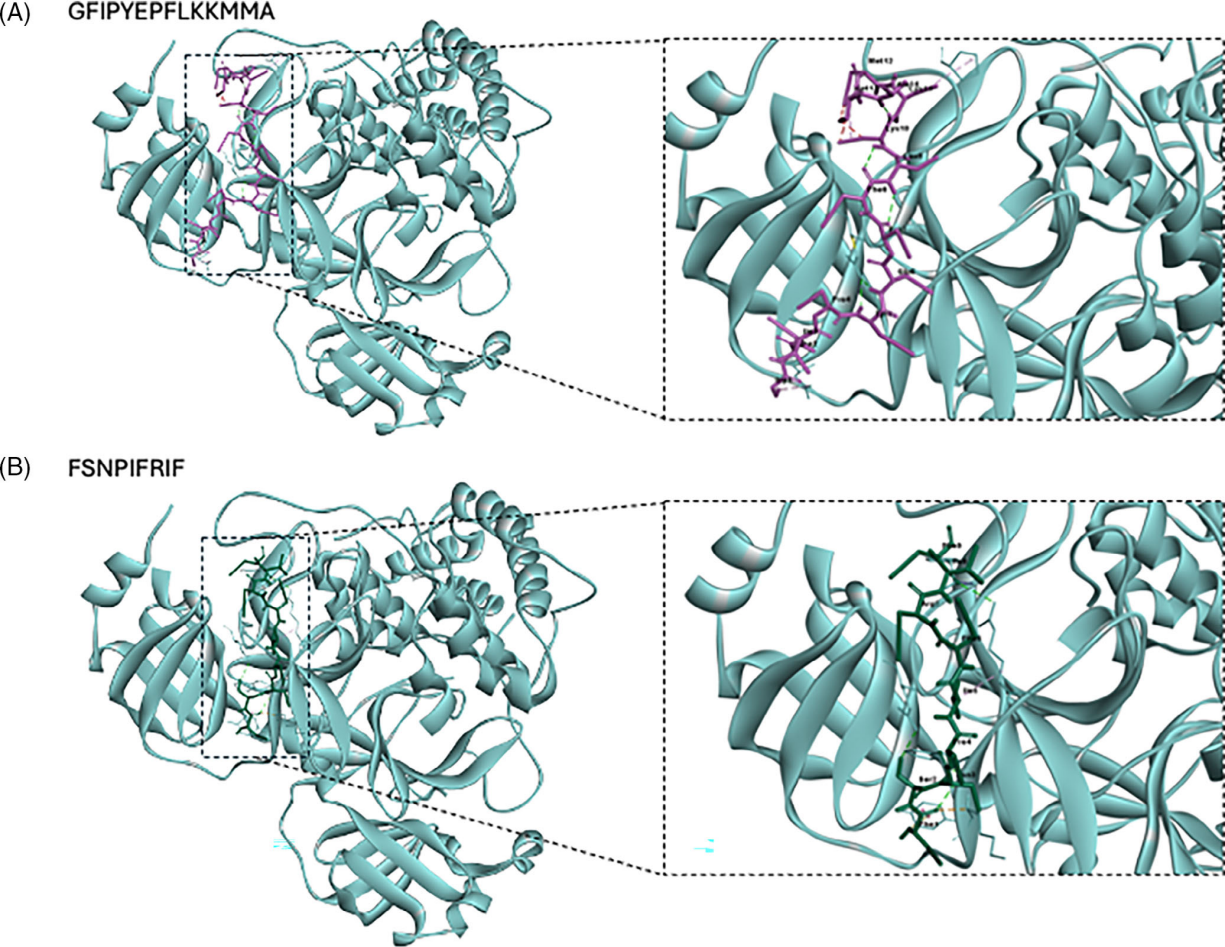
Visualization of the structures of receptor–peptide complexes using Biovia Discovery Studio Visualizer. (A) COVID19–GFIPYEPFLKKMMA binding site and interactions; and (B) COVID19–FSNPIFRIF binding site and interactions (PDB: 7C2Q).

**Figure 6:**
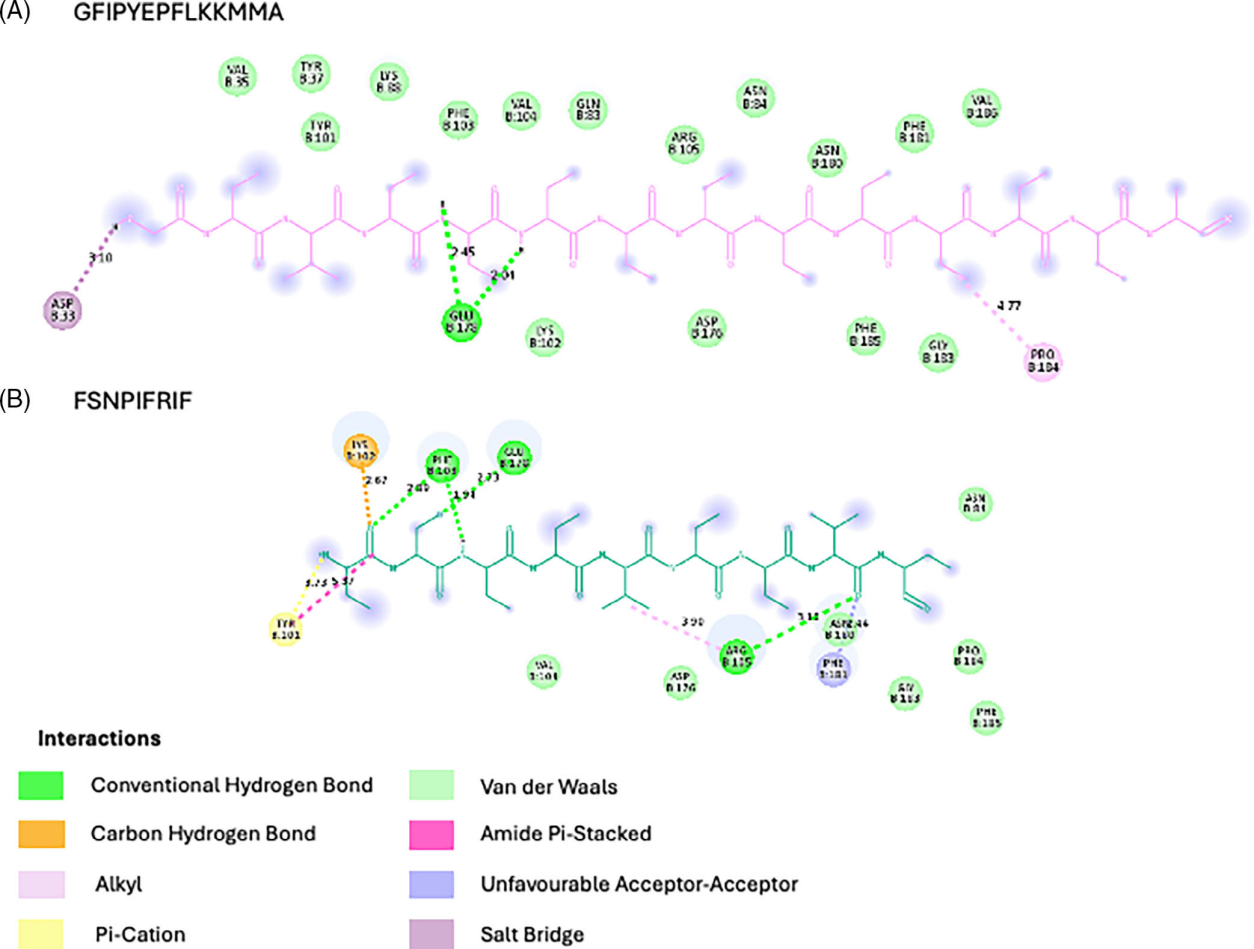
Analysis of 2D residue diagrams for selected peptides with their respective interaction bonds with COVID‐19 main protease in the apo state (PDB: 7C2Q) visualized using Biovia Discovery Studio Visualizer.

The peptides proposed as highly antiviral, according to the score, were GFIPYEPFLKKMMA and FSNPIFRIF (scores higher than 0.831). In antiviral peptide analyses, the focus was towards the COVID‐19 main protease in the apo state. The SARS‐CoV‐2 epidemic has been affecting the world since December 2019. According to reports, the variation of concern omicron had the greatest number of mutations, increasing its transmissibility and partially impairing its immunity caused by different vaccinations.^54^ The evaluation of peptides aims to evaluate the decrease of infection by SARS‐CoV‐2, and according to the values obtained (Table 3), both peptides could be considered as adequate antiviral components, especially GFIPYEPFLKKMMA with a value of −14.5 kcal mol^−1^. In Figs 5 and 6, the visualization of the interaction of each peptide with the COVID‐19 main protease in the apo state is depicted.

The results obtained were promising, as the affinity values reported are lower than those obtained by other authors recently, such as Bansal *et al*.,^55^ who reported among 10 peptides the lowest value of −6.4 kcal mol^−1^. In fact, recent studies sought to assess the potential of edible insects as a source of peptides against SARS‐CoV‐2 via *in silico* methods. Regarding this, Wong *et al*.^56^ found 82 sequences from the key proteins of three insects (mealworms, silkworm cocoons and housefly larvae), which were hypothesized to be absorbed by cells, and by using molecular docking to target the virus receptor‐binding domain, VPW was suggested as the most active peptide. Similarly, it was shown that PKWF and VHRKCF, identified from the *in silico* gastrointestinal digestion of mealworm proteins, were suitable peptides that may target the SARS‐CoV‐2 spike glycoprotein.^56^

##### Fungal receptors

**Figure 7:**
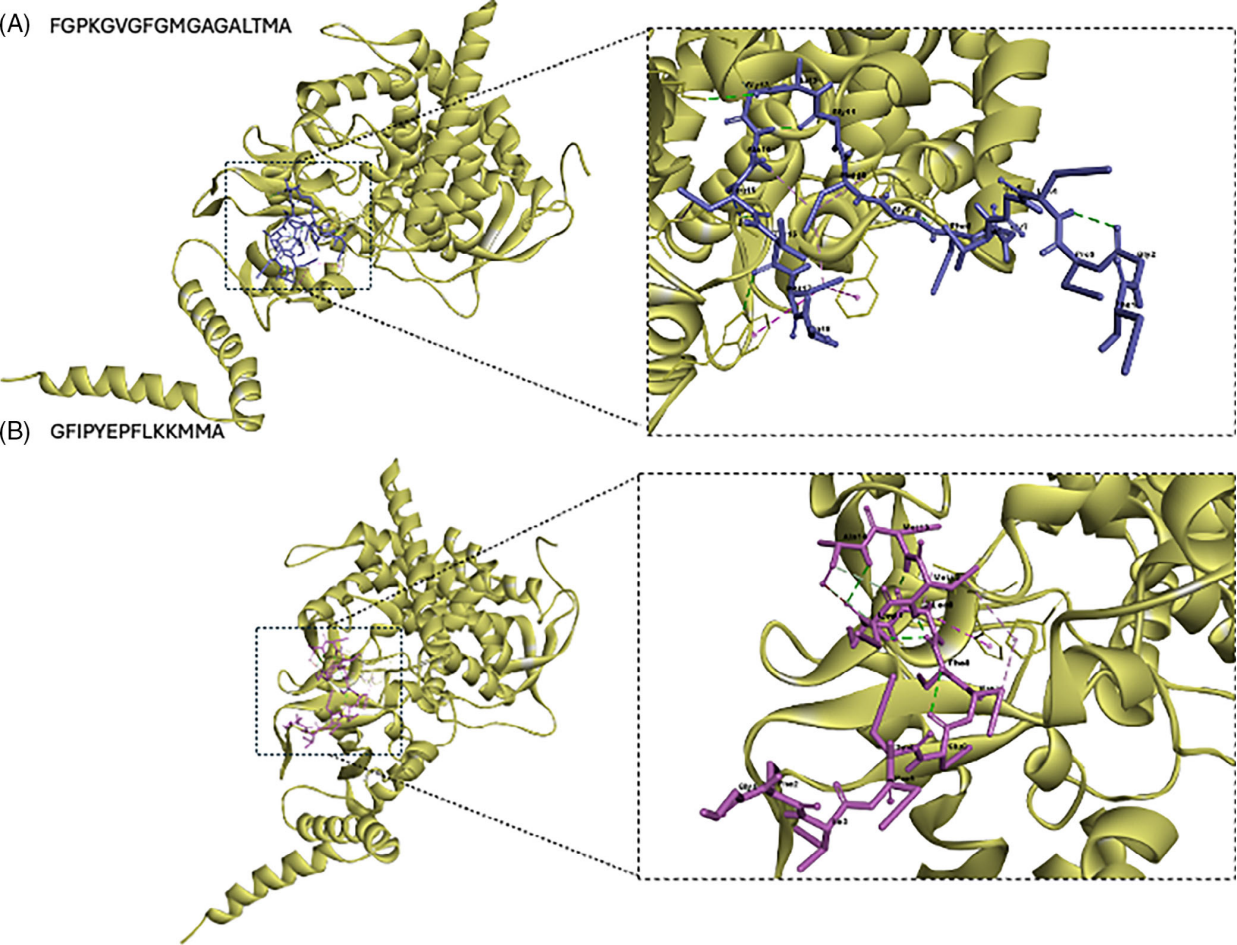
Visualization of the structures of receptor–peptide complexes using Biovia Discovery Studio Visualizer. (A) Lanosterol–FGPKGVGFGMGAGALTMA binding site and interactions; and (B) lanosterol–GFIPYEPFLKKMMA binding site and interactions (PDB: 4LXJ).

**Figure 8:**
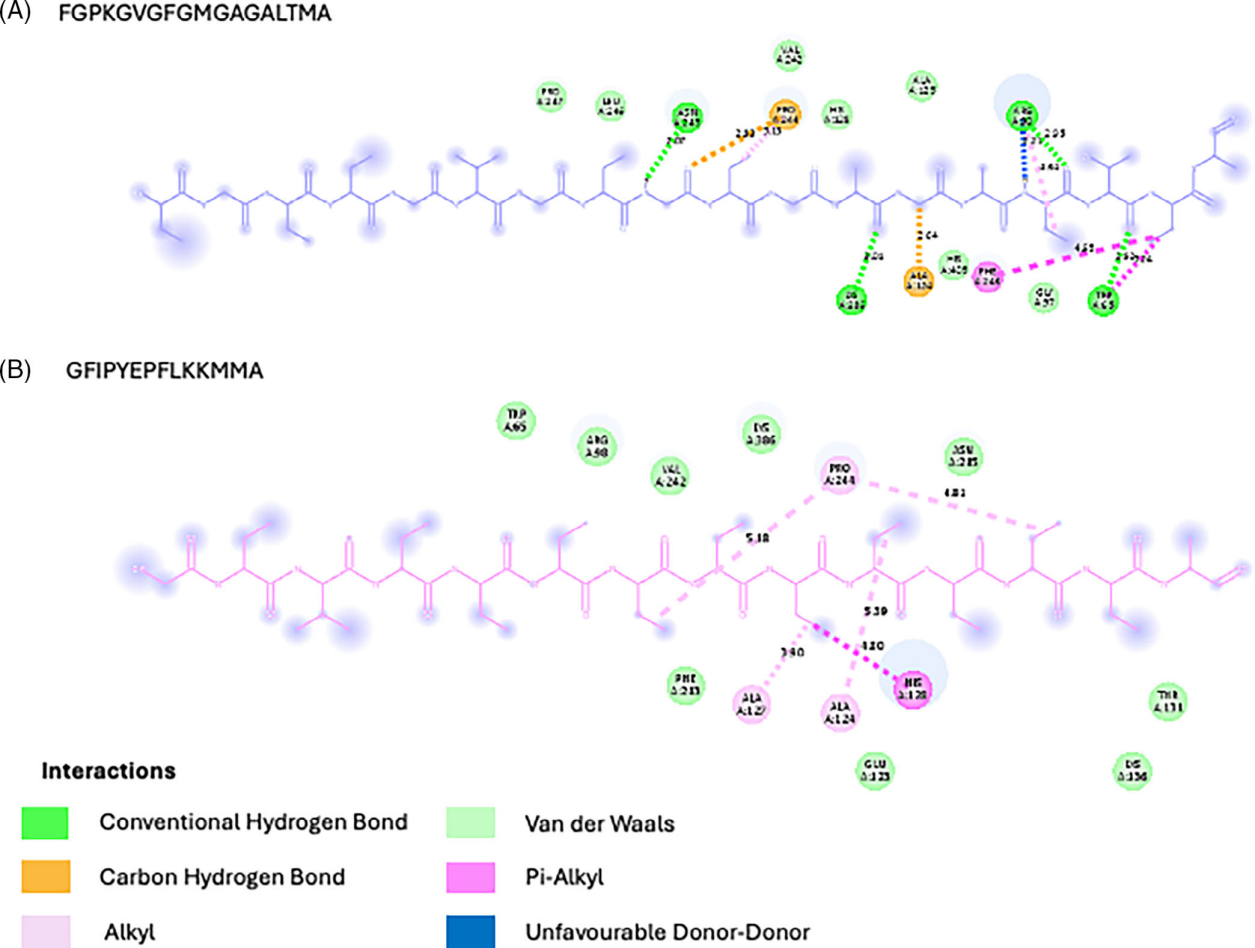
Analysis of 2D residue diagrams for selected peptides with their respective interaction bonds with the fungal receptor lanosterol 14‐*α* demethylase from *Saccharomyces cerevisiae* (PDB: 4XLJ) visualized using Biovia Discovery Studio Visualizer.

The peptides proposed as highly antifungal, according to the score, were GFIPYEPFLKKMMA and FGPKGVGFGMGAGALTMA (scores higher than 0.805). These two peptides were evaluated by molecular docking towards lanosterol 14‐*α* demethylase from *Saccharomyces cerevisiae* and the visualization of the interactions can be seen in Figs 7 and 8, whereas the molecular docking results are presented in Table 3. The relevance of lanosterol 14‐*α* demethylase relies on the fact that it is an enzyme playing a relevant role in the synthesis of ergosterol, which is an essential component of fungal cell membranes, and, consequently, the inhibition of the enzyme leads to a lack of this component, provoking the disruption of the membrane integrity and ultimately fungal cell death.^57^ In fact, *Saccharomyces cerevisiae*, despite being commonly considered safe for consumption and widely used in the food industry, is also known for producing different health consequences such as bloodstream infections (fungemia), pneumonia and infections at various other body sites, especially in patients with conditions such as HIV/AIDS, cancer and diabetes, or those undergoing organ transplantation.^58^

Both peptides had a high affinity energy, with values of −10 and −11.4 kcal mol^−1^ respectively for GFIPYEPFLKKMMA and FGPKGVGFGMGAGALTMA. These values show a similar affinity to the results reported by Tivari *et al*.,^59^ which correlated with an antifungal activity in some *in vitro* assays. Moreover, many atoms involved in the binding site on the receptor are the same in both studies, which supports the hypothesis of antifungal activity, although further studies should be carried out.

It must be noted that, even being that with higher affinity (less bioactive), GFIPYEPFLKKMMA was found to interfere with both viral and fungal receptors, thus indicating a multidisciplinary bioactivity in terms of antimicrobial activity.

To the authors’ knowledge, these identified peptides proposed as AMPs stemming from *T*. *molitor* protein hydrolysates have not been previously identified. The findings of this research pave the way for future experimental validations and optimizations. Further studies including *in vitro* analysis of the bioactive potential of the hydrolysate and the chemically synthesized peptides would be the next step to validate the results obtained. On top of that, evaluating how the degree of hydrolysis affects the AMPs’ presence in the sample could be also of interest, since the length of peptides with different activity highly varies. The applications that these protein and hydrolysates could have include the formulation of packaging (edible bioactive films) or the development of therapeutic strategies replacing antibiotics. The main problems with antibiotics, widely used to treat bacterial infection, are antibiotic resistance, potential side effects and disruption in the gut microbiota, provoking imbalance in the microbial community as well. Additionally, further refinements in the *in silico* approach can be explored to enhance accuracy and predictability, ultimately facilitating a more efficient screening process for potential AMPs.

## CONCLUSION

This research delved into the potential of *Tenebrio molitor* protein and an Alcalase‐derived hydrolysate as promising sources of AMPs through a groundbreaking *in silico* approach. Through the systematic exploration of physical–chemical features of the most active peptides based on prediction tools, together with the use of molecular docking analyses, novel peptides within the hydrolysates that exhibit notable antimicrobial properties were identified. The correlation between specific molecular characteristics and the efficacy of these peptides against various pathogens provides valuable insights into the design and development of novel antimicrobial agents. The *in silico* approach utilized in this study not only allows for a comprehensive analysis of a vast peptide landscape, but also offers a cost‐effective and time‐efficient means of screening potential candidates. The successful identification of bioactive peptides from *T*. *molitor* protein hydrolysates underscores the significance of exploring unconventional sources for bioactive compounds. As antibiotic resistance continues to pose a significant global threat, the discovery of alternative antimicrobial agents becomes increasingly imperative. This study contributes to the expanding knowledge base on unconventional sources of AMPs, while also emphasizing the potential of *in silico* methodologies in streamlining the bioactive compound discovery process. The insights gained from this research provide a solid foundation for the development of novel and effective antimicrobial agents.

## AUTHOR CONTRIBUTIONS

The authors' responsibilities were as follows: SMdlP and MJL designed research; EMP and TGdlR conducted research; EMP analysed data or performed statistical analysis; FRP wrote the paper; SMPdlP supervised the work. All authors have read and approved the final manuscript.

## FUNDING INFORMATION

This study was supported by research grant JDC‐2022‐050043 (Ministry of Science and Innovation, Government of Spain).

## CONFLICT OF INTEREST

The authors declare no conflict of interest.

## Supporting information


**Table S1.** Specific obtained correlation values.

## Data Availability

The data that support the findings of this study are available from the corresponding author upon reasonable request.
