# Supplementary material for: Exploring Tenebrio molitor as a source of low‐molecular‐weight antimicrobial peptides using a n in silico approach: correlation of molecular features and molecular docking
Source: J Sci Food Agric. 2024 Oct 16;105(3):1711–36. doi: 10.1002/jsfa.13949 (PMC11726611; doi:10.1002/jsfa.13949)
Supplement: Supplementary file 1 — Table S1. Specific obtained correlation values. [file JSFA-105-1711-s001.docx]

**Supplementary Table 1.** Specific obtained correlation values.

|  | Hydrophobicity | Steric hindrance | Sidebulk | Hydropathicity | Amphipathicity | Hidrophilicity | Net Hidrogen | Charge | pI | Mol. wt |
| --- | --- | --- | --- | --- | --- | --- | --- | --- | --- | --- |
| Antibacterial | 0.211 | 0.171 | 0.171 | 0.040 | -0.030 | -0.132 | -0.182 | 0.133 | 0.145 | 0.169 |
| Antiviral | 0.212 | 0.185 | 0.185 | 0.153 | 0.057 | -0.141 | -0.127 | 0.105 | 0.145 | 0.137 |
| Antifungal | 0.368 | 0.252 | 0.252 | 0.359 | -0.146 | -0.173 | -0.407 | 0.345 | 0.344 | 0.181 |
